# New and emerging treatments for schizophrenia: a narrative review of their pharmacology, efficacy and side-effect profile relative to established antipsychotics

**DOI:** 10.1016/j.neubiorev.2021.11.032

**Published:** 2021-11-24

**Authors:** Maria C. Lobo, Thomas S. Whitehurst, Stephen J. Kaar, Oliver D. Howes

**Affiliations:** 1 Institute of Psychiatry, Psychology and Neuroscience, King’s College London, London, UK; 2 MRC London Institute of Medical Sciences, Hammersmith Hospital, London, UK; 3 Institute of Clinical Sciences, Faculty of Medicine, Imperial College London, London, UK; 4 South London and Maudsley NHS Foundation Trust, Maudsley Hospital, London, UK; 5 H. Lundbeck UK, Ottiliavej 9, 2500 Valby, Denmark

**Keywords:** Schizophrenia, antipsychotic, cariprazine, brexpiprazole, lumateperone, pimavanserin, roluperidone, ulotaront, xanomeline, BI 425809, brilaroxazine, F17464

## Abstract

Schizophrenia is associated with substantial unmet needs, highlighting the necessity for new treatments. This narrative review compares the pharmacology, clinical trial data and tolerability of novel medications to representative antipsychotics. Cariprazine, brexpiprazole and brilaroxazine are partial dopamine agonists effective in acute relapse. Lumateperone (serotonin and dopamine receptor antagonist) additionally benefits asocial and depressive symptoms. F17464 (D3 antagonist and 5-HT1A partial agonist) has one positive phase II study. Lu AF35700 (dopamine and serotonin receptor antagonist) was tested in treatment-resistance with no positive results. Pimavanserin, roluperidone, ulotaront and xanomeline do not act directly on the D2 receptor at clinical doses. Initial studies indicate pimavanserin and roluperidone improve negative symptoms. Ulotaront and xanomeline showed efficacy for positive and negative symptoms of schizophrenia in phase II trials. BI 409306, BI 425809 and MK-8189 target glutamatergic dysfunction in schizophrenia, though of these only BI 425809 showed efficacy. These medications largely have favourable cardiometabolic side-effect profiles. Overall, the novel pharmacology, clinical trial and tolerability data indicate these compounds are promising new additions to the therapeutic arsenal.

## 1 Introduction

Schizophrenia is amongst the most disabling health conditions (Murray, C. J., Vos et al. 2012) and affects about 21 million people worldwide (Vos, Allen et al. 2016). People with schizophrenia have a life expectancy that is 15 years shorter than that of the general population, thought to be a result of increased rates of comorbid physical illness, increased smoking and drug use, reduced health-seeking behaviour as well as increased rates of suicide (Hjorthøj, Stürup et al. 2017, Pillinger, D’Ambrosio et al. 2019). Patients with schizophrenia experience positive, negative and cognitive symptoms affecting many aspects of their day-to-day functioning (McCutcheon, Robert A., Marques et al. 2020). Positive symptoms comprise hallucinations, delusions and thought disorder. Negative and cognitive symptoms include anhedonia, associality, avolition, alogia, blunted affect, inattention, poor executive functioning and working memory problems (Abi-Dargham 2014).

Subcortical dopamine dysfunction is thought to be a final common pathway to psychosis in many patients (Howes, Oliver D., Murray 2014). Supporting this, in vivo imaging studies have found large effect size increases in pre-synaptic dopamine synthesis and release in the striatum which are suspected to drive positive symptoms (McCutcheon, Robert, Beck, Jauhar, and Howes 2018a, Brugger, Angelescu et al. 2020a). Conversely, hypodopaminergia in the cerebral cortex has been linked to negative and cognitive symptoms (Slifstein, Van De Giessen et al. 2015). There is also evidence for the involvement of other neurotransmitter systems, in particular the serotonergic, cholinergic, and glutamatergic systems in the pathophysiology of schizophrenia (Kaar, Natesan et al. 2020, Selvaraj, Arnone et al. 2014).

Clinicians have a choice of over twenty licensed drug treatments to prescribe in schizophrenia (Christian, Saavedra et al. 2012, National Institute for Health and Care Excellence, (UK)). Nevertheless, current treatment is inadequate for a number of reasons. Firstly, current antipsychotics predominantly target positive symptoms, and do little for negative and cognitive symptoms (Leucht, Cipriani et al. 2013a). Secondly, antipsychotics are ineffective for many patients (Demjaha, Lappin et al. 2017). The majority of patients with schizophrenia started on medication will experience at least one relapse over the subsequent five years (Robinson, Woerner et al. 1999), and between a quarter and a third have treatment resistance (Demjaha, Lappin et al. 2017, Lally, Ajnakina et al. 2016). Finally, current drugs result in a range of distressing and sometimes disabling side effects (Kaar, Natesan et al. 2020) including extrapyramidal side effects, and metabolic side effects such as weight gain, hyperlipidaemia and type 2 diabetes, which may also contribute to lower life expectancy (Patel, Cherian et al. 2014, Pillinger, McCutcheon et al. 2020a).

Tailoring antipsychotic treatment to an individual is a key component of the long-term management of schizophrenia. The efficacy and tolerability of treatment in an individual both affect the likelihood of a patient continuing treatment and thus determine response and risk of relapse (Taylor, Barnes et al. 2018, Barnes, Drake et al. 2020). Adverse effects are a common reason for treatment discontinuation, particularly when patients do not see a benefit in their symptoms either (Taylor, Barnes et al. 2018).

Thus there is a need for new treatment options with improved efficacy and better tolerability, which are also effective for negative and cognitive symptoms. In recent years there have been a number of new treatments licensed, and several more are in late phase development. The purpose of this narrative review is to consider new additions to the available treatments for schizophrenia since 2015 as well as emerging medicines on the horizon. We aim to provide an update to help clinicians and researchers appraise where these new and emerging additions fit within the therapeutic arsenal. To this end, we review the clinically relevant pharmacology, clinical trial data and side-effect profile of these drugs and consider how they compare to established antipsychotics. We chose eight widely used antipsychotics as our reference group: haloperidol, chlorpromazine, amisulpiride, risperidone, olanzapine, quetiapine, aripiprazole and clozapine. We focus on highlighting the differences and potentially advantageous aspects of the new drugs relative to established antipsychotics.

## 2 Method

We aimed to review recent developments in the drug treatment for schizophrenia. Thus, we restricted the compounds we considered to agents that have either been approved by the United States Food and Drug administration and/or the European Medicines Agency since the beginning of 2015, or drugs in development for schizophrenia listed on clinicaltrials.gov with at least one phase II clinical trial published since the beginning of 2015.This identified the following compounds: cariprazine, brexpiprazole, lumateperone (or ITI-007), pimavanserin (or ACP-103), roluperidone (or MIN-101), ulotaront (or SEP-363856), xanomeline, Lu AF35700, BI 425809, BI 409306, brilaroxazine (or RP5063), F17464 and MK-8189. From May - October 2021 we then conducted a series of targeted literature searches in PubMed and clinicaltrials.gov for ‘schizophrenia’ paired with these psychopharmacological agents to identify published studies. Reference lists in publications identified by these searches were also checked to obtain any additional data sources. The websites of drug companies developing these medications were searched for press releases. Patent information was found by searching for the compound name in Google Scholar and including patents in the results. We selected phase II and phase III clinical trials assessing the efficacy and tolerability of these medications. We excluded trials evaluating patients with mental disorders other than schizophrenia. We extracted information on the pharmacology of each drug, reviewed all the clinical trial data and summarised the incidences of side-effects and adverse events. The evidence for each drug was evaluated according to the GRADE (Grading of Recommendations, Assessment, Development and Evaluations) framework (Siemieniuk, Guyatt 2019) and an overall assessment of the quality of evidence was made.

## 3 Cariprazine

### 3.1 Pharmacology

**Figure 1:**
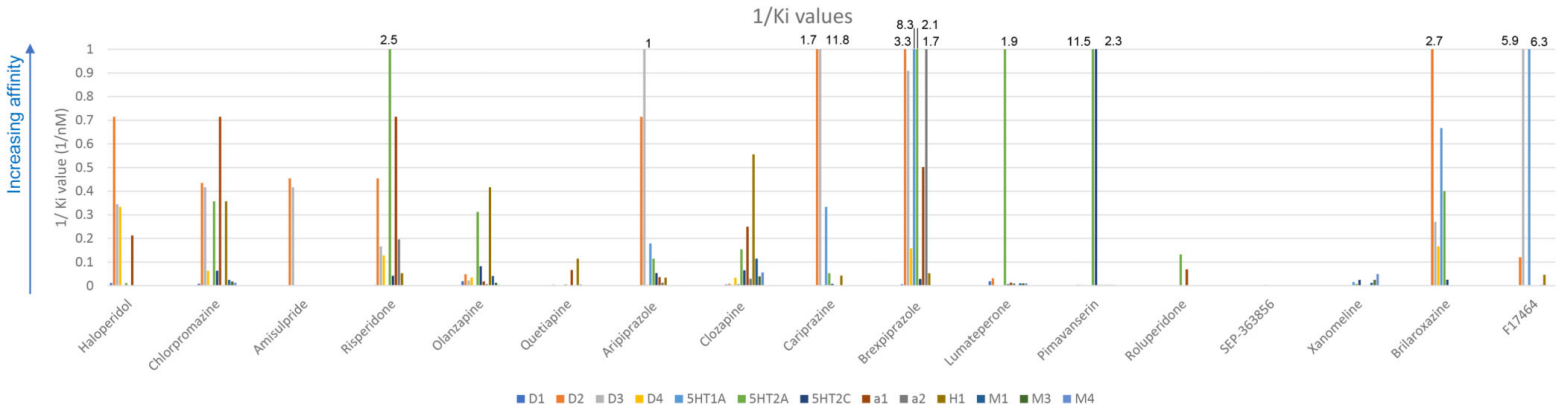
1/Ki values for new and representative established antipsychotics. Established antipsychotics are all bind to D2 receptors, whereas several newer antipsychotics have no appreciable affinity for D2 receptors.

**Figure 2:**
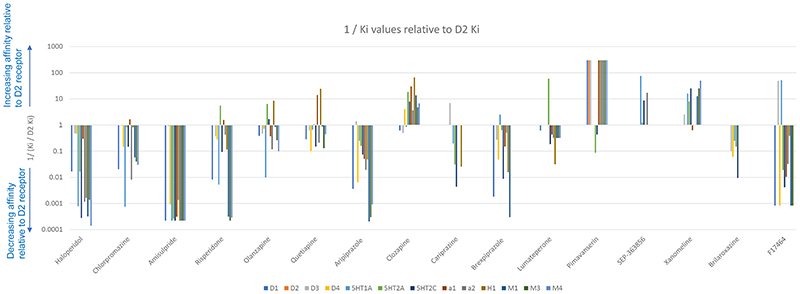
1/ Ki values relative to D2 Ki for new and well-established antipsychotics. There is wide variation between antipsychotics in their affinities for other receptors relative to their affinities for the D2 receptor.

**Figure 3:**
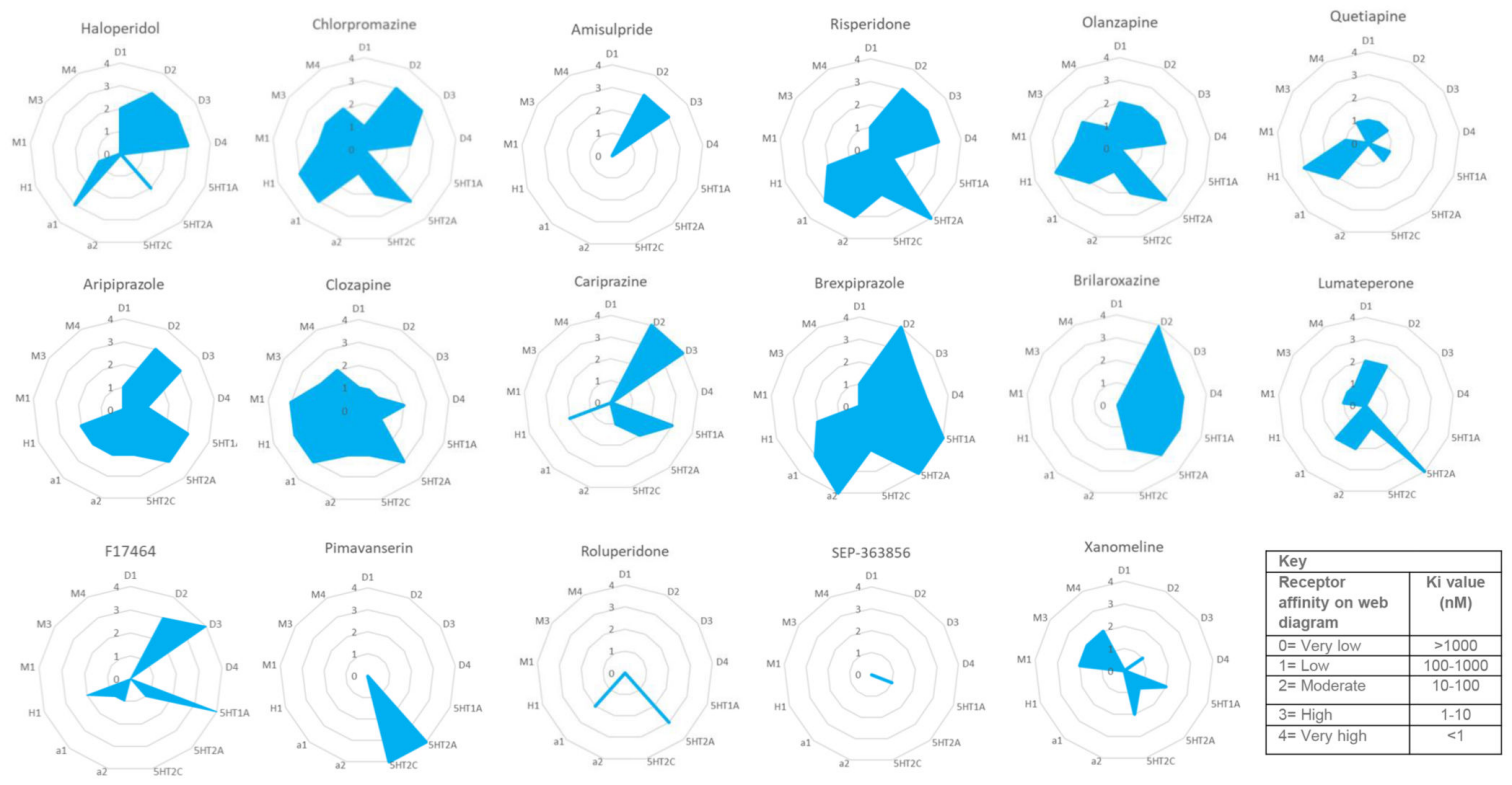
Web diagrams showing receptor affinities for new and well-established antipsychotics. Each antipsychotic has a distinct receptor binding profile. The numbers on the concentric lines represent the affinity (Ki) of a drug for that receptor where 4 = very high affinity (Ki <1 nM), 3 = high affinity (Ki 1-10 nM), 2 = moderate affinity (Ki 10 – 100 nM), 1 = low affinity (Ki 100 – 1000 nM) and 0 = very low affinity (Ki > 1000 nM).

Cariprazine is a partial agonist at dopamine D2 and D3 receptors, with an almost seven-fold greater affinity for D3 relative to D2 receptors (K_i_= 0.085nM and 0.59nM respectively, see Table 1 and Figure 2 (Corponi, Serretti et al. 2017, Citrome 2013)). This distinguishes cariprazine from the majority of other antipsychotics, which have low or negligible D3 receptor affinity (see Figures 1 and 3). Of note, blonanserin also has a high affinity for D3 (K_i_=0.494nM), although this is still lower than cariprizine’s, and blonanserin has a higher affinity for D2 than cariprazine (K_i_=0.142 nM) (Tenjin, Miyamoto et al. 2013). It has been proposed that cariprazine’s high affinity for D3 receptors relative to D2 could be effective for negative and cognitive symptoms (Krause, Zhu et al. 2018). Cariprazine has similar intrinsic agonist activity at the D2 receptor compared to aripiprazole, though higher intrinsic agonist activity at D3 (Tadori, Forbes et al. 2011, Kiss, Horváth et al. 2010).

Cariprazine is also a partial agonist at 5-HT1A receptors (K_i_=3.0nM), although this is one-fifth affinity of its affinity for D2 receptors (Citrome 2013), and is an antagonist at 5-HT2B receptors with similar affinity (K_i_=0.58nM) to its affinity for D2 receptors (Kiss, Horvath et al. 2010). It is also an antagonist at 5HT2A and H1 receptors, albeit with affinities for these receptors that are between 1/10 and 1/100th that of its affinity for D2 (Citrome 2013). Cariprazine has very low affinity (less than 1/100 of its affinity for D2) for 5HT2C and α1 receptors, meaning it is likely to have negligible occupancy of these at clinical doses (Citrome 2013).

Following administration of a single dose of cariprazine, peak plasma concentration is reached within 3-6 hours (Garnock-Jones 2017). Cariprazine is metabolised hepatically by cytochrome P450 3A4 (CYP3A4) and cytochrome P450 2D6 (CYP2D6) enzymes to two equipotent metabolites, desmethyl cariprazine and didesmethyl cariprazine (Reyad, Mishriky 2019). After repeated doses, steady state is reached between weeks 1 and 2 for cariprazine and desethyl cariprazine and between weeks 4 and 8 for didesmethyl cariprazine (Garnock-Jones 2017). The elimination half-life of cariprazine and desmethyl cariprazine is 2-5 days and of didesmethyl cariprazine is 2-3 weeks (Garnock-Jones 2017, Reyad, Mishriky 2019).

### 3.2 Clinical efficacy

There have been four placebo-controlled randomised controlled trials (RCTs) of cariprazine to date in 2172 patients in total (Table 2). All tested use of cariprazine or placebo for a treatment period of 6 weeks in patients experiencing an acute relapse of schizophrenia.

Three of these studies (Durgam et al 2015, Kane et al 2015 and Durgam et al 2014) found a statistically significant improvement in Positive and Negative Syndrome Scale (PANSS) with cariprazine compared to placebo (Durgam, Cutler et al. 2015, Kane, Zukin et al. 2015, Durgam, Starace et al. 2014).

The Durgam et al 2016 study did not find a significant difference in outcome between low and high dose cariprazine groups and the placebo group after correction for multiple comparisons. However, without correction for multiple comparisons the low dose cariprazine group showed significantly greater reduction in PANSS total and PANSS negative scores compared to placebo (Durgam, Litman et al. 2016).

Meta-analyses estimate the mean difference in change from baseline in PANSS total score for patients taking cariprazine compared to placebo is between -6.23 and -9.71 following 6 weeks of treatment ((Cooper, Mishriky et al. 2020, Zhao, Qin et al. 2018, Corponi, Serretti et al. 2017), an improvement of similar magnitude to that seen with other antipsychotics on meta-analysis (Mizuno, McCutcheon et al. 2020, Leucht, Cipriani et al. 2013b). This amelioration in symptoms is clinically significant, with more patients on cariprazine shifting to an improved category on the Clinical Global Impression (CGI) scale after treatment compared to those on placebo (Durgam, Earley et al. 2017).

Two RCTs included an arm with another antipsychotic as an active comparator. In the Durgam et al 2014 study, all cariprazine groups (1.5-4.5mg) and the risperidone 4mg group showed a significant improvement in PANSS score over placebo, with the cariprazine groups resulting in a less marked mean change in PANSS compared to risperidone (Durgam, Starace et al. 2014). In the Durgam et al 2015 study, cariprazine 3mg, 6mg and aripiprazole 10mg were all superior to placebo with a statistically significant improvement in PANSS. The cariprazine 6mg group had a numerically greater change in PANSS compared to aripiprazole 10mg, though this difference was not tested for statistical significance (Durgam, Cutler et al. 2015).

One active controlled trial assessed the effect of cariprazine on negative symptoms. Nemeth et al randomised 461 patients with long-term stable schizophrenia and predominant negative symptoms to receive cariprazine 4mg or risperidone 4mg for 26 weeks. The cariprazine group had a statistically significant greater improvement in PANSS-factor score for negative symptoms compared to the risperidone group (mean difference -1.46, p 0.002) (Németh, Laszlovszky et al. 2017). The low absolute difference (-1.46) in PANSS-factor score for negative symptoms does raise the question of whether this is a clinically meaningful difference. Nevertheless, on the basis of this trial, the European Medicines Agency has included an indication of the efficacy of cariprazine for treatment of predominantly negative symptoms of schizophrenia on its license (European Medicines Agency). Moreover, two observational studies of open label cariprazine have found a clinically significant improvement in negative symptoms in patients treated with cariprazine in real-life settings (Smulevich, Ivanov et al. 2020, Rancans, Dombi et al. 2021). Nevertheless, there is a need for further controlled trials of the effect of cariprazine on negative symptoms as there has been only one randomised controlled trial specifically investigating this question to date. Due to the complexity of negative symptoms and their assessment, it is also important to note the limitations of PANSS and other scales in evaluating negative symptoms in clinical trials and the need for better instruments (Blanchard 2006, Kirkpatrick 2006, Hopkins 2018).

Corponi et al carried out a network meta-analysis of the efficacy of cariprazine on overall symptoms, positive and negative symptoms and quality of life, extracting the results from four randomised controlled trials of cariprazine and comparing them with those reported for other antipsychotics in previous meta-analyses (Corponi, Serretti et al. 2017). Cariprazine was ranked 14^th^ out of 16 antipsychotics for overall efficacy, 7^th^ out of 10 for effect on positive symptoms and 5^th^ out of 11 for effect on negative symptoms, ranking higher than aripiprazole in this last domain. As part of this meta-analysis, a meta-regression was additionally carried out to identify the impact of clinical and demographic variables on response to cariprazine. This found that a high baseline PANSS score, shorter duration of disease and younger age were associated with greater response to cariprazine (Corponi, Serretti et al. 2017), in line with findings for other antipsychotics (Howes, Oliver D., Whitehurst et al. 2021).

There has been one long-term study looking at the efficacy of cariprazine in preventing relapse. 765 patients were enrolled to receive open-label cariprazine (3, 6 or 9mg) for 20 weeks (Durgam, Earley et al. 2016). 200 patients who completed this treatment and were then eligible to continue in the next phase of the study were then randomised to either continue cariprazine or receive placebo in the double-blind treatment phase of up to 72 weeks. For those continuing on cariprazine, mean time from baseline to first symptom relapse was 224 days compared to 92 days for the placebo group (Durgam, Earley et al. 2016).

Two studies investigating cariprazine use in children and adolescents are due to read out in the next few years.

### 3.3 Side effect profile

In the pooled data from 8 short- and long-term clinical trials of cariprazine, the most commonly reported adverse events were akathisia (14.6%), insomnia (14%) and headache (12.1%) (Barabássy, Sebe et al. 2021). The incidence of extrapyramidal disorder was 7.0% (Barabássy, Sebe et al. 2021). Sedation and somnolence affected 3.7% and 3.1% of subjects respectively (Barabássy, Sebe et al. 2021). Overall, cariprazine has been characterised as a predominantly activating antipsychotic, where rates of akathisia and restlessness are higher than rates of sedation or somnolence (Citrome 2017). Nevertheless, akathisia is generally of mild to moderate severity and >93% of study participants experiencing akathisia were able to continue cariprazine (Barabássy, Sebe et al. 2021). The majority of cases of akathisia also responded to anti-extrapyramidal symptom medication or drug down-titration (Barabássy, Sebe et al. 2021).

In terms of metabolic effects, cariprazine has been associated with a small increase in weight (mean increase of 1.58 kg over 48 weeks) (Nasrallah, Earley et al. 2017). Levels of total cholesterol, high-density lipoprotein (HDL) and fasting triglycerides decreased from baseline following initiation of cariprazine (Barabássy, Sebe et al. 2021). Increases in fasting glucose were small and same as placebo (mean change of 0.3 mmol/L) (Barabássy, Sebe et al. 2021). Cariprazine stands out as the only antipsychotic associated with a significant reduction in low-density lipoprotein (LDL) cholesterol in short-term placebo controlled trials (Pillinger, McCutcheon et al. 2020a). Cariprazine is not associated with hyperprolactinaemia (Barabássy, Sebe et al. 2021, Earley, Durgam et al. 2017, Lao, He et al. 2016). There are very few incidences of QT prolongation with cariprazine, with 0.4% of patients from the pooled tolerability data reporting QTc >500 msec (Barabássy, Sebe et al. 2021). Szatmári et al extracted data from two clinical studies including 49 adolescents and 17 elderly patients and found that cariprazine was generally safe and well-tolerated in the these populations and that frequencies of adverse events were similar to the adult population (Szatmári, Barabássy et al. 2020).

With regard to serious adverse events (SAEs), Earley et al observed in their pooled analysis of safety and tolerability data that the most commonly reported SAE was worsening of schizophrenia (2.8% of cariprazine group versus 5% of placebo groups) (Earley, Durgam et al. 2017). In these data, two deaths occurred. Both were in the cariprazine 6mg group; one a suicide and the other classified as an ischaemic stroke/myocardial infarction. Neither death was considered related to treatment. Overall, suicidal ideation and suicidality treatment-emergent adverse events were similar for cariprazine and placebo groups (3.6 vs 4.7% and 0.4% vs 0.2% respectively) (Earley, Durgam et al. 2017). The more recent pooled analysis by Barabássy et al found rates of SAEs were lower among the cariprazine group compared to placebo (2.7% vs 3.1%) but the specific causes of SAEs were not defined further (Barabássy, Sebe et al. 2021). Barabássy et al found that suicidality was more common in the cariprazine group over placebo, though <1% overall (0.9% vs 0.1%) (Barabássy, Sebe et al. 2021).

### 3.4 Summary

Cariprazine has been shown to result in clinically significant improvement in patients experiencing an acute relapse of schizophrenia, though on a network meta-analysis uses indirect comparisons its ranking relative to other antipsychotics in terms of overall efficacy was relatively low. One maintenance study showed its efficacy in delaying time to relapse over placebo, but further long-term studies are needed to investigate this. There is some evidence that cariprazine is particularly effective in treating negative symptoms which may be linked to its high affinity for D3 receptors, though further testing in this group is warranted. Cariprazine may also be of benefit to patients with irregular medication compliance, due to the long half-life of its active metabolite didesmethyl cariprazine. Cariprazine is an activating antipsychotic with relatively higher rates of akathisia and restlessness than other newer antipsychotics, and is thus recommended for morning dosing. It has minimal metabolic side effects and would be a good choice for patients with metabolic risk factors.

## 4 Brexpiprazole

### 4.1 Pharmacology

Brexpiprazole is a dopamine partial agonist belonging to the same class as aripiprazole and cariprazine (Demyttenaere, Detraux et al. 2019). It has a greater affinity for D2 receptors (Ki=0.3nM) and lower affinity for D3 receptors (Ki=1.1nM) compared to both aripiprazole and cariprazine (Roth, Lopez). Brexpiprazole also has a greater affinity for D1 and D4 receptors (Ki=164 and 6.3 nM respectively) than aripiprazole as shown in Table 1 and Figure 1. There are no data for the affinity of cariprazine for these receptors, precluding comparison.

The intrinsic agonist activity of aripiprazole at D2 is reported at 25-90% of that of dopamine, depending on assay-specific factors including receptor density (Burris, Molski et al. 2002). In one assay directly comparing the two antipsychotics, brexpiprazole demonstrated intrinsic agonist activity at D2 of 43% of that of dopamine, whereas for aripiprazole this was 61% (Maeda, Sugino et al. 2014). Thus, although it has greater affinity for D2, brexpiprazole has less intrinsic agonist activity at this receptor compared to aripiprazole.

Brexpiprazole is a partial agonist at 5HT1A receptors and antagonist at 5-HT2A and α1 receptors (Demyttenaere, Detraux et al. 2019, Kishi, Ikuta et al. 2020). With the exception of 5HT2C, brexpiprazole binds to serotonin, alpha adrenergic and histamine receptors with greater affinity than aripiprazole and cariprazine (notwithstanding the lack of data for cariprazine’s affinity for α1 and α2 receptors, see Table 1 and Figures 1 and 3) (Roth, Lopez, Stahl 2017, Nerkar, Bhise).

Peak plasma concentration of brexpiprazole is attained 4 hours after a single dose administration. Steady-state concentrations after repeated dosing are reached in 10-12 days (Mauri, Paletta et al. 2018). Like cariprazine, brexpiprazole is metabolised by CYP3A4 and CYP2D6 enzymes, though its major metabolite, DM-3411, does not contribute to its therapeutic effects. The elimination halflife of brexpiprazole is 3-4 days (Mauri, Paletta et al. 2018).

### 4.2 Clinical efficacy

There have been five short-term randomised placebo-controlled trials of brexpiprazole to date, with a total of 2683 patients (Table 3). All investigated brexpiprazole’s efficacy in patients experiencing an acute relapse of schizophrenia for a treatment period of six weeks, with two studies also including a treatment arm with an alternative antipsychotic (quetiapine or aripiprazole) as an active control. The primary outcome measure in all five short-term studies was change in PANSS score from baseline.

Three of the studies found that the brexpiprazole group had a greater reduction in PANSS score compared to placebo, though this difference only reached statistical significance for specific brexpiprazole dose groups in each trial (2mg in the study by Ishigooka et al (Ishigooka, Iwashita et al. 2018), 2mg and 4mg in the ‘Vector’ study (Correll, Skuban et al. 2015), and 4mg in the ‘Beacon’ study (Kane, Skuban et al. 2015)). The other doses of brexpiprazole tested in these studies did lead to a numerically greater reduction in PANSS score over placebo but did not reach statistical significance.

A further study by Correll et al did not find a significant difference in change in PANSS between the brexpiprazole treatment groups and the placebo group, but the aripiprazole active control group also failed to separate from placebo (Correll, Skuban et al. 2016). In the ‘Lighthouse’ study (NCT01810380), the brexpiprazole group failed to separate from the placebo group but also had a statistically significant poorer outcome at the end of the study compared to the quetiapine group (Marder, Hakala et al. 2017). There was notably a high placebo response in the Lighthouse study, and additionally further analysis found that functional unblinding of patients experiencing typical side effects of quetiapine may have been a confounding factor resulting in its better performance over brexpiprazole (Marder, Eriksson et al. 2020).

When the data of the Vector, Beacon and Lighthouse studies were analysed together, brexpiprazole 2-4mg was found to result in a statistically significant improvement in PANSS score over placebo (treatment difference -5.8, p<0.0001) (Marder, Hakala et al. 2017). Post-hoc analysis of these three studies also showed that the brexpiprazole group had a greater mean reduction in PANSS particularly among the more severely ill patients (Meade, Shi et al. 2020).

Additional work has further investigated the efficacy of brexpiprazole relative to other antipsychotics. Citrome et al 2016 performed a randomised open-label study testing use of brexpiprazole or aripiprazole over 6 weeks, and did not find a significant difference in reduction in PANSS score between either antipsychotic (Citrome, Ota et al. 2016). Moreover, a recent network meta-analysis of thirty-two oral antipsychotics ranked brexpiprazole 31st (out of 32 drugs) for overall change in symptoms, 21st (out of 21) for effect on positive symptoms and 18th (out of 21) for effect on negative symptoms (Huhn, Nikolakopoulou et al. 2019). Brexpiprazole performed relatively better with respect to effect on social functioning and was ranked 6th out of the 13 antipsychotics where this was measured (Huhn, Nikolakopoulou et al. 2019).

The ‘Equator’ maintenance study investigating treatment over 52 weeks concluded that brexpiprazole significantly delayed time to relapse relative to placebo (Fleischhacker, Hobart et al. 2017). Two further open label extension studies found that brexiprazole 1-4mg was associated with mean reductions in PANSS score of -12.2 and -6.8 respectively (Forbes, Hobart et al. 2018, Hakala, Gislum et al. 2018).

Table 3 shows five studies currently in progress for brexpiprazole which are due to complete in the next few years. They are investigating brexpiprazole use in particular patient groups, including adolescents and comorbid substance users, as well as further exploring the incidence of adverse events.

### 4.3 Side-effect profile

In the 52-week open label study of 1072 patients by Forbes et al, the most common treatment-emergent adverse events were insomnia (8.6%), weight gain (7.8%), headache (6.4%) and agitation (5.4%) (Forbes, Hobart et al. 2018). The second open label study with a smaller sample size of 210 patients by Hakala et al also found similar rates of these adverse events (Hakala, Gislum et al. 2018). Brexpiprazole has almost half the incidence of akathisia compared to aripiprazole (5.5% compared to 10.0%) (Forbes, Hobart et al. 2018), and, furthermore, a lower incidence than cariprazine (Demyttenaere, Detraux et al. 2019).

A network meta-analysis of the metabolic effects of antipsychotics determined brexpiprazole, alongside aripiprazole, to be associated with the best metabolic outcomes and one of the agents of choice for patients with an increased risk of developing metabolic complications (Pillinger, McCutcheon et al. 2020b). Brexpiprazole is associated with a small increase in weight (mean increase 2.1kg over 52 weeks) (Forbes, Hobart et al. 2018). Nevertheless, there is no associated effect on glucose, total cholesterol, LDL, triglycerides or QTc interval for brexpiprazole compared to placebo (Huhn, Nikolakopoulou et al. 2019, Pillinger, McCutcheon et al. 2020b). Brexpiprazole is not associated with sustained hyperprolactinaemia (Ivkovic, Lindsten et al. 2019).

There were six deaths in the safety and tolerability data for 2315 patients on brexpiprazole pooled by Kane et al, but none of these were considered related to the medication (Kane, Skuban et al. 2016, Forbes, Hobart et al. 2018). In short term studies, rates of treatment-emergent adverse events (TEAEs) related to suicidality were the same for brexpiprazole and placebo groups (0.2%), and in long term studies, 0.8% of patients on brexpiprazole reported a TEAE related to suicidality (Kane, Skuban et al. 2016).

### 4.4 Summary

Brexpiprazole has had mixed results with respect to its clinical efficacy in clinical trials to date, with some doses not separating from placebo in some trials. However, in one of these trials the comparator antipsychotic also failed to separate, suggesting the trial may have been sub-optimal to test efficacy. Brexpiprazole has a relatively favourable side effect profile; it is less activating than aripiprazole, and has minimal metabolic effects. Studies in progress will provide further information about its efficacy, long-term tolerability and role in treating specific patient sub-groups.

## 5 Brilaroxazine (RP5063)

### 5.1 Pharmacology

Brilaroxazine (RP5063) has a chemical structure that is similar to aripiprazole, and acts as a high-affinity partial agonist on D2, D3 and D4 receptors (Ki= 0.37nM, 3.7nM and 6.0nM respectively), and on serotonin 5-HT1A and 5-HT2A receptors (Ki= 1.5nM, 2.5nM respectively) (Cantillon, Prakash et al. 2017). Brilaroxazine also has antagonistic activity on serotonin 5-HT2B, 5-HT2C, 5-HT6 and 5-HT7 receptors (Ki= 0.19nM, 39nM, 51nM and 2.7nM respectively) and has moderate affinity for the serotonin transporter (Ki=107nM) (Cantillon, Prakash et al. 2017).

Brilaroxazine has a half-life of over 40 hours allowing once-daily dosing, with steady-state reached after 8 days of administration (Cantillon, Prakash et al. 2017).

### 5.2 Clinical Efficacy

A phase II randomised controlled trial of brilaroxazine in acute relapse of schizophrenia or schizoaffective disorder found that the brilaroxazine 15mg and 50mg groups showed statistically significant reductions in PANSS total score compared to the placebo group (Table 4) (Cantillon, Prakash et al. 2017). The brilaroxazine 30mg arm did not show statistically significant difference relative to placebo, which the authors reported may have been due to a larger number of drop-outs in this group for reasons not related to medication. The active control group, aripiprazole 15mg, also failed to separate from placebo, though the authors commented that this group had solely been included for sensitivity analysis and not as a comparator (Cantillon, Prakash et al. 2017). PANSS subscale scores showed greater brilaroxazine improvement versus placebo in PANSS negative and prosocial symptoms than positive symptoms (Cantillon, Prakash et al. 2017). There are currently no further clinical trials of brilaroxazine registered on clinicaltrials.gov, though the authors of the published phase II study report that they intend to initiate two further phase III trials (Cantillon, Bhat 2018).

### 5.3 Side effect profile

In NCT0149008, commonly reported treatment emergent adverse events (occurring in ≥ 2% of any brilaroxazine group and not reported for placebo) included extrapyramidal symptoms, akathisia, and raised liver enzymes, though >95% of these were mild or moderate in severity (Cantillon, Prakash et al. 2017). Among 170 subjects receiving brilaroxazine, there were 4 serious treatment emergent adverse events (all of which were elevated liver enzymes). Additionally in the brilaroxazine group, 1 subject had a grand mal seizure and there was 1 case of epileptiform discharges on EEG (Cantillon, Prakash et al. 2017). There were no significant changes to BMI, serum glucose, prolactin or ECG parameters in the brilaroxazine group (Cantillon, Prakash et al. 2017). Maintenance studies are needed to assess the long-term efficacy and tolerability of brilaroxazine.

### 5.4 Summary

Brilaroxazine is a partial agonist on D2, D3 and D4 receptors as well as serotonin 5HT1A and 5-HT2A receptors with a long half-life which may be helpful for patients with intermittent compliance. One phase II study found that brilaroxazine 15mg and 50mg groups showed statistically significant improvement in PANSS score relative to placebo, though the 30mg group did not. Brilaroxazine is associated with a low incidence of extrapyramidal symptoms and no metabolic effects, though further long-term studies are needed.

## 6 Lumateperone (ITI-007)

### 6.1 Pharmacology

Lumateperone (ITI-007) was selected from a series of compounds tested for their suitability as potential new antipsychotics because of its very high affinity for serotonin 5-HT2A receptors and additional interactions with dopamine receptors and serotonin transporters (Davis, Robert E., Correll 2016).

Lumateperone is a 5-HT2A antagonist, with a 60-fold higher affinity for 5-HT2A receptors compared to D2 receptors (Ki values of 0.54nM and 32 nM respectively; see Figure 2) (Snyder, Vanover et al. 2015). At clinical antipsychotic doses, lumateperone is therefore likely to result in near total blockade of 5-HT2A receptors (Davis, Robert E., Correll 2016).

It has been suggested that lumateperone could act as an agonist at presynaptic dopamine D2 autoreceptors to reduce dopamine synthesis whilst also acting as an antagonist at postsynaptic dopamine D2 receptors. Supporting this hypothesis, Snyder et al found that lumateperone demonstrates postsynaptic D2 antagonism and presynaptic D2 partial agonism in the striatum of mice (Snyder, Vanover et al. 2015). In contrast, Zhang and Hendrick carried out agonist and antagonist assays of lumateperone and other antipsychotics in Chinese Hamster Ovary (CHO) cells expressing recombinant human D2L and D2S receptors (Zhang, Hendrick 2018), and found that lumateperone demonstrated full antagonism at both D2L and D2S, with no demonstrable agonism at either receptor. In vivo, D2L receptors are found in postsynaptic dopamine responsive cells, and the D2S receptor (a splice variant of D2L hypothesised to be the autoreceptor) is most abundant on presynaptic dopamine cells (Usiello, Baik et al. 2000). These data thus question whether lumateperone does show presynaptic D2 agonism, although the authors acknowledged that the G-proteins found in CHO cells do not replicate those found in human presynaptic cells (Zhang, Hendrick 2018). Notwithstanding this, further work is needed to test if lumateperone does show agonism at presynaptic D2 receptors.

N-methyl-D-aspartate (NMDA) receptor hypofunction is thought to contribute to the pathophysiology of schizophrenia (Kokkinou, Irvine et al. 2020a). Lumateperone has been found to increase NMDA receptor activity in mesolimbic regions by increasing the phosphorylation of the GluN2B subunit (Corponi, Fabbri et al. 2019, Snyder, Vanover et al. 2015). Thus, in addition to its actions on the dopamine system, lumateperone could help address glutamatergic dysfunction in the disorder (McCutcheon, Robert A., Krystal et al. 2020). Additionally, lumateperone inhibits serotonin reuptake via inhibition of serotonin transporters (with a moderate affinity, Ki=62 nM), which could also contribute to its therapeutic action (Davis, Robert E., Correll 2016).

Lumateperone binds to a number of other receptors to a lesser degree. It has moderate affinity for α1, α2 and 5-HT2C receptors (Ki=73, <100 and 173 nM respectively) and low affinity for M3 and H1 receptors (Ki>100nM and >1000nM respectively, see figures 1 and 3). Whether lumateperone has agonist or antagonist activity at these receptors is not clear to date (Meyer 2020, Snyder, Vanover et al. 2015, Blair 2020).

A PET study of patients with schizophrenia receiving lumateperone 60mg daily found that the mean peak dorsal striatal D2 receptor occupancy of lumateperone was 39%, a lower receptor occupancy level than most currently licensed antipsychotic drugs at their efficacious doses (Vanover, Kimberly E., Davis et al. 2019).

Lumateperone is available as oral capsules of the compound lumateperone tosylate.

Lumateperone tosylate 60mg contains 42mg of the active moiety lumateperone (U.S. Food and Drug Administration 2019). Following oral administration, lumateperone is rapidly absorbed and reaches peak plasma concentration in 3-4 hours. The drug is metabolised by CYP3A4 of the cytochrome P450 system (Davis, Robert E., Correll 2016). Lumateperone has a half-life of 13 hours and lumateperone’s metabolites, IC200161 and IC200131, have half-lives of 20 and 21 hours respectively (Davis, Robert E., Correll 2016). The metabolites IC200161 and IC200131 are both active and have a similar pharmacologic profile to lumateperone (Davis, Robert E., Correll 2016). ICI200131 can also be metabolically transformed by CYP3A4 back to lumateperone, resulting in sustained plasma lumateperone levels (Davis, Robert E., Correll 2016).

### 6.2 Clinical efficacy

Lumateperone was first approved in December 2019 in the USA for the treatment of adults with schizophrenia (Blair 2020). There have been three randomised controlled trials of lumateperone to date in 1481 patients in total (Table 5). All investigated lumateperone’s use in patients with an acute relapse of schizophrenia over a treatment period of 4-6 weeks.

Two of the RCTs (ITI-007-005 and ITI-007-301) had positive findings, though in both trials only the group receiving the 60mg dose of lumateperone tosylate showed a significantly greater improvement in PANSS score relative to the placebo group. The lumateperone tosylate 120mg group in ITI-007-005 and lumateperone tosylate 40mg group in ITI-007-301 both failed to separate from placebo (Lieberman, Davis et al. 2016, Correll, Davis et al. 2020).

The authors of the ITI-007-005 study suggested the lack of efficacy for total symptoms with lumateperone tosylate 120mg may have been the result of increased sedation masking signs of clinical improvement (Lieberman, Davis et al. 2016). However, in this study there was also no significant change relative to placebo in the PANSS positive subscale for the lumateperone tosylate 120mg group. As change in positive symptoms may be expected to be less sensitive to sedation, this could suggest that other factors also contributed to the lack of significant differences.

In study ITI-007-302, neither lumateperone tosylate 20mg nor 60mg separated from placebo, while the active control (risperidone 4mg) did (Vanover, Kimberly, Dmitrienko et al. 2018). Nevertheless, a high placebo response rate was observed in this trial, which could have reduced the power of the study to detect effects in the active arms.

In further analysis, study ITI-007-005 found that lumateperone tosylate 60mg significantly reduced scores on the PANSS-derived prosocial factor, which assessed difficulties with social functioning (Lieberman, Davis et al. 2016). Similarly, the results of study ITI-007-301 indicated that patients taking lumateperone tosylate 60mg demonstrated improved social functioning compared to placebo (Correll, Davis et al. 2020). With regards to depressive symptoms, an a priori defined subgroup analysis in study ITI-007-005 found that lumateperone tosylate 60mg significantly reduced the Calgary Depression Scale for Schizophrenia in the 7 patients in this arm with symptoms of depression (Lieberman, Davis et al. 2016). This fits with an expected antidepressant effect due to its inhibition of serotonin reuptake and could suggest a role for lumateperone in schizophrenia with co-morbid depression, although this requires further testing in a much larger study.

The results of two additional open label studies investigating the pharmacokinetics and tolerability of lumateperone long acting injection and of oral lumateperone in adolescents are awaited.

### 6.3 Side effect profile

In a pooled analysis of short-term placebo controlled trials (n=818), the most common adverse events in patients receiving lumateperone tosylate 60mg were sedation (24%), nausea (9%), dry mouth (6%), dizziness (5%), increased creatine phosphokinase (4%), fatigue (3%), vomiting (3%), increased hepatic transaminases (2%) and decreased appetite (2%). In these trials the incidence of extrapyramidal symptoms was 6.7% with lumateperone and 6.3% with placebo (U.S. Food and Drug Administration 2019). In the short-term studies, weight gain occurred in 2% of lumateperone groups compared to 3% of placebo (corresponding mean weight change of +1.6kg and +1.3kg) (Kane, Vanover et al. 2020). A more recent open-label tolerability study of 301 outpatients found the incidence of extrapyramidal symptoms to be 1.0%, lower than the aforementioned pooled analysis (Correll, Vanover et al. 2021).

There has been one longer term open-label safety study in 602 patients, which showed that lumateperone was well tolerated over 1 year (Satlin, A., Vanover et al. 2019). In the long-term study, lumateperone was associated with significant reductions from baseline in mean body weight, BMI and waist circumference (Satlin, Andrew, Durgam et al. 2020).

Like cariprazine and brexpiprazole, lumateperone has a favourable cardiovascular profile, with no QTc interval prolongation (Davis, Robert, Dmitrienko et al. 2018). In the 12 month open-label study, total cholesterol, LDL and prolactin levels decreased when patients were switched from their standard antipsychotic treatment to lumateperone (Satlin, A., Vanover et al. 2019). Post-hoc analysis of studies ITI-007-005, ITI-007-302 and ITI-007-303 showed that patients on lumateperone had reduced rates of metabolic syndrome at the end of both the short term and long term studies (Edwards, Satlin et al. 2021).

Two patients on lumateperone in the ITI-007-301 study experienced severe treatment emergent adverse events that led to them discontinuing treatment; one was orthostatic hypotension and the other convulsions (in a patient with pre-existing risk factors and a history of seizures) (Correll, Davis et al. 2020). There were no deaths in the RCTs in the lumateperone group and no increase in suicidal ideation or behaviour.

### 6.4 Summary

Lumateperone’s pharmacology is characterised by high-affinity 5HT2A antagonism, inhibition of serotonin transporters and relatively low striatal D2 receptor occupancy compared to other antipsychotics at therapeutic doses (McCutcheon, Robert A., Marques et al. 2020). Two of three RCTs investigating its efficacy have shown positive results in total symptoms over placebo, with some indication that it may also improve social functioning and depressive symptoms.

Lumateperone appears to have a favourable tolerability profile, with rates of extrapyramidal symptoms similar to placebo and no significant metabolic side effects detected in short and long-term studies, though a quarter of patients in short-term studies experienced sedation. Further efficacy and safety trials are required, especially long-term studies, and there has yet to be a relapse prevention study.

## 7 F17464

### 7.1 Pharmacology

F17464 is a D3-antagonist and 5-HT1A partial agonist (Cosi, C., N’Guyen et al. 2017). Like cariprazine, F17464 has very high affinity for D3 receptors (Ki=0.17nM) and 5-HT1A receptors (Bitter, I., Groc et al. 2017, Cosi, C., N’Guyen et al. 2017). F17464 exhibits high affinity for D2 receptors (Ki=9.3nM) where it acts as a very weak partial agonist (Cosi, C., N’Guyen et al. 2017).

A PET-scan study in healthy volunteers showed that F17464 has >80% D3 receptor occupancy with little (<20%) D2 receptor occupancy at 15 and 30mg (Slifstein, Abi-Dargham et al. 2020).

Evidence suggests there is restricted distribution of the D3 receptor in the brain and that it plays a role in limbic brain functions by modulating glutamatergic pathways (Sokoloff, Le Foll 2017). The D3 antagonism of F17464 could potentially improve cognition by addressing low dopamine tone in the prefrontal cortex, while partial agonism at 5-HT1A could also be associated with pro-cognitive effects (Bitter, I., Groc et al. 2017).

Maximum plasma concentration of F17464 is reached 0.5 – 4 hours after oral administration (Slifstein, Abi-Dargham et al. 2020). Steady-state is reached after 15 days (Bitter, Istvan, Lieberman et al. 2019). The PET study indicated that while the mean plasma half-life of F17464 is 1.32 hours, the drug remains detectable at D3 receptors 22 hours post-dose, making it suitable for twice-daily dosing (Slifstein, Abi-Dargham et al. 2020).

### 7.2 Clinical Efficacy

There has been one short-term phase II double blinded RCT of F17464 (Table 6), which found a statistically significant improvement in PANSS score for the F17464 20mg BD group over placebo (Bitter, Istvan, Lieberman et al. 2019). Secondary efficacy analyses also found a statistically significant effect of F17464 on PANSS positive score, but no difference in PANSS negative score or Marder negative factor (Bitter, Istvan, Lieberman et al. 2019). Nevertheless, the study population (acute relapse of schizophrenia) and short duration of the study meant the trial was unlikely to have adequately tested a potential effect on negative symptoms. Post-hoc analysis in this study using the Wallwork factors of the PANSS items did suggest a beneficial effect of F17464 on cognitive ability (Bitter, Istvan, Lieberman et al. 2019). Of note, the authors stated that 10 randomised patients had been excluded from the manuscript due to the breach of Good Clinical Practice standards by one study centre. There were also major protocol deviations for a further 19 subjects, whose data was analysed in the full analysis set, but not in the per protocol set (Bitter, Istvan, Lieberman et al. 2019).

### 7.3 Side effect profile

The most common treatment-emergent adverse events in NCT02151656 with higher incidence in F17464 than placebo were insomnia (10.4%), agitation (7.5%), hyperlipidaemia (7.5%) and akathisia (4.5%) (Bitter, Istvan, Lieberman et al. 2019). All serious adverse events in this study were lack of efficacy-related adverse events (14.9% on F17464 and 22.4% on placebo). 13 patients (19.4%) in the F17464 arm discontinued treatment, which was due to treatment inefficacy (11 patients), raised liver enzymes (1 patient) and suicidal ideation (1 patient) (Bitter, Istvan, Lieberman et al. 2019).

No patients taking F17464 reported extrapyramidal symptoms and F17464 did not lead to a clinically relevant change in ECG parameters. F17464 was associated with an increase in prolactin levels during the study and hyperprolactinaemia was more frequent and more marked in females (Bitter, Istvan, Lieberman et al. 2019).

### 7.4 Summary

F17464 is a high-affinity D3-antagonist and 5-HT1A partial agonist. One phase II study showed an improvement in PANSS score in the F17464 40mg group relative to placebo, with possible beneficial effect on cognition suggested from post-hoc analysis. F17464 is an activating antipsychotic with insomnia, agitation and akathisa some of the most common side effects. It is associated with hyperprolactinaemia.

## 8 Lu AF35700

### 8.1 Pharmacology

Lu AF35700 is an antagonist of D1, 5-HT2A and 5-HT6 receptors (Fellner 2017). A Positron Emission Tomography study has demonstrated Lu AF35700 has predominant D1 vs D2 dopamine receptor occupancy combined with high 5-HT6 receptor occupancy (NIH U.S. National Library of Medicine 2020a).

### 8.2 Clinical efficacy

There have been two clinical trials of Lu AF35700 in schizophrenia so far, both in treatment-resistance (Table 7). In both cases, treatment resistance was determined by a history of non-response to adequate prior antipsychotic treatment and failure of a prospective trial of either olanzapine or risperidone, after which non-responders were then randomised to either Lu AF35700 or to continue on the drug they were currently taking (olanzapine or risperidone). The ‘Anew’ study (NCT03230864) aimed to test Lu AF35700 in patients who had developed treatment resistance early or late in their illness. However, the Lu AF35700 10mg group was not significantly different from the risperidone/olanzapine group, though the study was likely under-powered as it achieved less than 50% of its patient recruitment targets (NIH U.S. National Library of Medicine 2020c). The ‘Daybreak’ study (NCT02717195) was much larger than the Anew study, enrolling over 1000 patients and recruiting over 200 patients to each double-blinded arm. It tested Lu AF35700 at doses of 10mg and 20mg. Whilst both groups showed reductions in PANSS total scores and improvements on other measures neither group performed significantly better than the active control (olanzapine/risperidone) group on the primary outcome of change in PANSS total score, or secondary outcomes (NIH U.S. National Library of Medicine 2019). Whilst these results show no significant advantage of Lu AF35700 over olanzapine/risperidone for treatment resistant schizophrenia, it remains unclear if it is efficacious in schizophrenia because there was no placebo control group.

### 8.3 Side effect profile

In the Daybreak study, the most common adverse events with Lu AF35700 were headache (4.6%-6.47% with Lu AF35700 10-20mg, compared to 3.4% with risperidone and 4.1% with olanzapine) and weight increase (3.4% with Lu AF35700 10mg, 8.2% with Lu AF35700 20mg vs 4.8% with olanzapine/risperidone) (NIH U.S. National Library of Medicine 2019). The Anew study found a lower incidence of weight increase with Lu AF35700 10mg compared to continued olanzapine/risperidone (2.86% vs 9.09%) (NIH U.S. National Library of Medicine 2020c). No statistical testing was done to assess the statistical significance of these differences. There is no data regarding effects of Lu AF35700 on ECG parameters or prolactin.

The incidence of akathisia was <3% in both studies and was similar for Lu AF35700 and olanzapine/risperidone groups. In the Daybreak study, somnolence occurred in 1.3% of the Lu AF35700 10mg and 3.9% of Lu AF35700 20mg groups (compared to 1.8% with olanzapine/risperidone), while rates of somnolence were not reported in the Anew study (NIH U.S. National Library of Medicine 2019, NIH U.S. National Library of Medicine 2020c). There were no deaths among the patients taking Lu AF35700 (NIH U.S. National Library of Medicine 2019, NIH U.S. National Library of Medicine 2020c).

A maintenance study of open-label Lu AF35700 10-20mg/day over 57 weeks (NCT02892422) with results on clinicaltrials.gov (though not yet published) found that headache was the most common adverse event, affecting 8.2% of subjects. The incidence of other non-serious adverse events was not reported. 28 of 524 subjects (5.3%) experienced a serious adverse event; worsening of schizophrenia (21 subjects), suicide attempt (2), intentional overdose (2), myocardial infarction (1), pulmonary embolism (1), hip fracture (1), coma (1), parkinsonism (1), alcohol withdrawal (1), disinhibition (1) and insomnia (1). There were no fatalities (NIH U.S. National Library of Medicine 2020b).

### 8.4 Summary

Lu AF35700 is a D1, 5HT2A and 5HT2 receptor antagonist. There have been two clinical trials of Lu AF35700 in treatment resistance, neither of which found significant improvement with Lu AF35700 over olanzapine or risperidone active controls. Lu AF35700 was generally well-tolerated with the most common side effect reported being headache, and some evidence of an association with weight gain at the higher dose of 20mg.

## 9 Pimavanserin (ACP-103)

### 9.1 Pharmacology

Pimavanserin (ACP-103) is already licensed for psychosis in Parkinson’s disease in the United States of America, and is now being trialled for schizophrenia. Unlike the novel treatments discussed thus far, pimavanserin has minimal affinity for the dopamine receptor (Ki>1000nM, see Table 1). It has high affinity for the 5HT_2A_ receptor (K_i_=0.087nM, Table 1), where it is thought to act as an inverse agonist (ACADIA Pharmaceuticals Inc 2016). Interestingly, this high affinity for 5HT2A is similar to that of clozapine, which shows good efficacy in treating psychosis in Parkinson's disease (Hacksell, Burstein et al. 2014). Pimavanserin also has high affinity for 5HT2C receptors (K_i_=0.44nM) and appreciable, but much lower affinity for sigma-1 receptors (K_i_=120nM), and negligible affinity (K_i_ >1000nM) at 5-HT1A, 5-HT2B, histamine, muscarinic, and alpha receptors (ACADIA Pharmaceuticals Inc 2016) (see Figures 1 and 3), suggesting low risk of side-effects such as sedation and constipation due to actions at these receptors.

The half-life of pimavanserin is 57 hours with peak concentration occurring at 6 hours (Kitten, Hallowell et al. 2018), and the half-life of its chief metabolite is about 200 hours (Baltzersen, Meltzer et al. 2020). A positron emission tomography study demonstrated that 10mg of Pimavanserin was sufficient to occupy over 90% of brain 5HT_2A_ receptors in healthy volunteers (Nordstrom, Mansson et al. 2008). Pimavanserin is metabolised by CYP 450 enzymes, and plasma pimavanserin levels decrease in combination with inducers such as rifampicin and increase in combination with inhibitors of this system (Kitten, Hallowell et al. 2018).

### 9.2 Clinical Efficacy

The initial studies of pimavanserin were in Parkinson’s disease psychosis, and the Food and Drug Administration approved its use in this condition in 2016, a decision upheld on review in 2018 (Meltzer, Mills et al. 2010). There was some controversy around the approval, since two trials failed to show a significant benefit relative to placebo (Hacksell, Burstein et al. 2014, Kitten, Hallowell et al. 2018, Webster 2018) and the study showing a significant benefit used a previously untested rating scale. Pimavanserin has subsequently been tested in schizophrenia, where it was well tolerated compared to placebo when added on to treatment as usual (Table 8) (Abbs, Bugarski-Kirola et al. 2020).

A press release reporting results from a phase-II clinical trial indicated no significant benefit of add-on treatment with pimavanserin on total PANSS score or clinical global impression (CGI), but a significant benefit on change in negative symptom scores relative to placebo (ACADIA Pharmaceuticals Inc 2019a). However, this statement reported an unadjusted p-value of 0.047, which is unlikely to survive correction for multiple statistical tests (ACADIA Pharmaceuticals Inc 2019a). A press release from a phase-III trial of pimavanserin as an add-on to usual antipsychotic treatment reported that there was no significant difference in total PANSS scores or CGI (ACADIA Pharmaceuticals Inc 2019b). There were significant benefits of add-on primavanserin in the PANSS negative symptoms subscale and PANSS Marder negative factor score (unadjusted p values 0.047 and 0.034 respectively), but it was not reported whether the results for these secondary outcomes would survive correction for multiple comparisons (ACADIA Pharmaceuticals Inc 2019b). Results are available on the National Library of Medicine Clinical Trials Database (ACADIA Pharmaceuticals Inc 2020a, ACADIA Pharmaceuticals Inc 2020b) and data relating to safety and adherence has been published in abstracts (Abbs, Bugarski-Kirola et al. 2020, Bugarski-Kirola, Nunez et al. 2020) but a full peer-reviewed report is not yet available for either trial.

Three further studies in schizophrenia are ongoing and due to read out in the next three years. One study currently recruiting is a randomised placebo-controlled trial assessing the effect of adjunctive pimavanserin on negative symptoms (NCT04531982). The remaining two studies are open label; a year-long open-label study to assess tolerability (NCT03121586), and a study aiming to correlate treatment response to pimavanserin monotherapy and positron emission tomography measurements of 5HT2_A_ occupancy (NCT03994965, Baltzersen, Meltzer et al. 2020).

### 9.3 Side effect profile

Pimavanserin did not worsen motor symptoms in Parkinson’s disease (Meltzer, Mills et al. 2010) and, similar to brexpiprazole and lumateperone, had low propensity to cause extrapyramidal side effects in schizophrenia (Bugarski-Kirola, Nunez et al. 2020). Pimavanserin is not associated with a rise in serum prolactin (Abbas, Roth 2008). There is a black box warning for increased risk of death when used in the elderly, similar to the recent warnings for other antipsychotics (Baltzersen, Meltzer et al. 2020). Pimavanserin increased QT by 7.2ms on average in a group of Parkinson’s patients over 40, and manufacturers suggest caution when co-prescribing with other drugs with QT-prolonging potential and avoiding use in patients with established QT prolongation (Markham 2016). In Parkinson’s disease trials, more patients suffered from the following adverse events in the pimavanserin group relative to the placebo group: nausea, peripheral oedema, confusion, hallucinations, constipation and gait disturbance, with a prevalence between 1 and 7% (Markham 2016, Cummings, Isaacson et al. 2014).

### 9.4 Summary

Pimavanserin stands out due to its combination of high affinity for 5HT_2A_ receptors, where it is a partial agonist, and low affinity for D2 receptors. Trial data in Parkinson’s disease indicate it has a minimal propensity to cause extra pyramidal side effects. It has been studied as an adjunct to antipsychotics in patients with schizophrenia with no effect on total PANSS scores, but possible beneficial effects on negative symptoms, although these findings have not been subject to peer review.

## 10 Roluperidone (MIN-101)

### 10.1 Pharmacology

According to both a patent application and the main article for a recent phase-II trial, roluperidone (MIN-101) is a high affinity 5-HT2A antagonist (K_i_= 8.19 nM), sigma 2 receptor antagonist (K_i_ = 7.53nM, and has some action as an alpha1-adrenergic antagonist (Luthringer, Pellegrini et al. 2013, Davidson, Saoud et al. 2017). However, detailed methods are not reported, including whether these binding assays were conducted on human brain tissue (see Table 1 and Figures 1 and 3) (Davidson, Saoud et al. 2017, Luthringer 2016, Luthringer, Pellegrini et al. 2013). Roluperidone has been reported to have low or no affinity for muscarinic, cholinergic, and histaminergic receptors, although, again, the data or methods used have not been reported (Davidson, Saoud et al. 2017).

The compound has a half-life of around 6 hours (Luthringer, Pellegrini et al. 2013). Phase I and II trials have shown that once daily dosing at 32 or 64mg is sufficient to attain a plasma concentration similar to the concentration required to elicit antipsychotic like action in rats (Luthringer, Pellegrini et al. 2013). Steady state was achieved after 7 days at dose of 1mg/kg in rats (Luthringer, Pellegrini et al. 2013). There are two main metabolites, which are reported to have similar binding profiles and similar half-lives to roluperidone. However, one metabolite had an affinity several orders of magnitude greater than roluperidone at guinea pig histamine receptors (K_i_ of 43.6nM) (Luthringer 2016, Ebdrup, Bjørn H., Rasmussen et al. 2011).

### 10.2 Clinical Efficacy

The first phase II study showed no significant benefit for roluperidone over placebo on total PANSS scores (Table 9) (Ebdrup, Bjørn H., Rasmussen et al. 2011). However, a second phase II trial showed significantly greater improvement for both 32mg and 64mg roluperidone relative to placebo on the primary outcome of endpoint of change in PANSS negative symptom factor, as well as for total PANSS score, at 12 weeks (Davidson, Saoud et al. 2017, Marder, Davis et al. 1997). Patients in the high-dose group also showed a significant improvement at 12 weeks relative to placebo in a composite cognitive score measured with the Brief Assessment of Cognition in Schizophrenia (Keefe, Harvey et al. 2018). There were significant roluperidone-associated improvements in specific domains of cognitive functioning, such as visuospatial reasoning and verbal memory (Keefe, Harvey et al. 2018).

A phase-III trial has completed recruitment, although we await a peer-reviewed publication of the results (Minerva Neurosciences Inc 2020). According to a recent presentation, there was a significant benefit of both high and low doses over placebo at 4 weeks on PANSS negative symptom factor, but this was only significant for the high dose at 8 weeks, and there was no significant effect of either dose at 12 weeks, which was the primary endpoint (Minerva Neurosciences Inc 2020). The presentation draws attention to a larger placebo effect in the phase III trial compared to the phase II trial, and to the fact that when they used the raw subscale scores for negative symptoms as an end-point, the significant benefits of high-dose treatment were sustained at 12 weeks (Minerva Neurosciences Inc 2020).

### 10.3 Side effect profile

Headache (7.5% vs 3.6%), asthenia (5.6% vs 2.4%), and somnolence (3.7% vs 0%) were more common in the treatment than placebo group (Davidson, Saoud et al. 2017). Not surprisingly, given the higher rates of somnolence, insomnia was less common in the treated groups compared to placebo. Side effect data was not available from the larger phase III trial (Minerva Neurosciences Inc 2020). A potential issue identified on the patent application is an increased risk of QTc prolongation with plasma concentrations over 80ng/mL, but clinical trials have so far not reported any instances of significant QTc prolongation at therapeutic doses (Luthringer, Pellegrini et al. 2013). Two patients in the phase II trial suffered serious adverse events other than relapse in schizophrenia symptoms; one had vomiting and abdominal pain and another reported syncope and bradycardia (Davidson, Saoud et al. 2017). In contrast to current antipsychotics, roluperidone has no major effects on prolactin levels or extrapyramidal side effects relative to placebo (Davidson, Saoud et al. 2017).

### 10.4 Summary

Encouraging effects of roluperidone on negative symptoms of schizophrenia found in initial phase II trials appear not to have been fully replicated in a larger study, although the secondary analyses may indicate an effect. The strength of these findings is not clear as the peer reviewed report has not yet been published. Roluperidone is generally well tolerated, and does not show propensity to cause extrapyramidal side effects, but detailed information on side effects from the largest study is not yet available.

## 11 Ulotaront (SEP-363856)

### 11.1 Pharmacology

Ulotaront (SEP-363856) is a novel antipsychotic distinctive for its agonist activity at trace amine associated receptor 1 (TAAR1; EC50=0.14μM, maximum efficacy=101.3%) (Dedic, Jones et al. 2019). An in vitro receptor screen against a panel of neuroreceptors found that ulotaront is also an agonist at the 5HT1A receptor, although its affinity is an order of magnitude lower than that for the TAAR1 (EC50=2.3μM, maximum efficacy=74.7%), and that it has lower affinity for 5-HT2A and D2 receptors (K_i_=17250 and 21300 nM respectively), where ulotaront acts as a weak partial agonist (Dedic, Jones et al. 2019) (see Table 1 and Figures 1 and 3). Further in vivo testing in rat and primate brains showed that ulotaront did not produce significant D2 receptor occupancy at clinically relevant doses (Dedic, Jones et al. 2019). Therefore, any therapeutic effects are likely to be independent of a direct action on D2 receptors.

TAAR1 agonism has an inhibitory effect on the firing of dopaminergic and serotonergic neurons (Revel, Moreau et al. 2011). Further mouse studies show that ulotaront inhibits neuron firing in the ventral tegmental area of the midbrain, as well as inhibiting dorsal raphe neuronal activity via 5-HT1A agonism (Dedic, Jones et al. 2019). Elevated striatal dopamine synthesis capacity is thought to be a key component of the pathophysiology of schizophrenia (McCutcheon, Robert, Beck et al. 2018, Brugger, Angelescu et al. 2020), and this can be reproduced in a ketamine mouse model (Kokkinou, Irvine et al. 2020b). A recent study in this ketamine mouse model shows ulotaront reduces the ketamine-induced increases in striatal dopamine synthesis capacity (Kokkinou, Irvine et al. 2020b). This suggests ulotaront may target this aspect of the pathophysiology of the disorder, in contrast to current second-generation antipsychotics (Jauhar, Veronese et al. 2019). Overall, the preclinical and pharmacological studies to date indicate that while ulotaront does not act directly on D2 receptors in vivo, it inhibits dopaminergic neuron activity, probably predominantly through TAAR1, although 5HT1A agonism may also contribute to its effects.

### 11.2 Clinical efficacy

There has been one randomised placebo-controlled phase II trial to date. This involved 245 participants with an acute relapse of schizophrenia. This study found a significant improvement in the ulotaront group over placebo, with a mean difference in PANSS score of -7.5 (p<0.01) over the 4-week treatment period (Table 10) (Koblan, Kent et al. 2020). The placebo response (mean change in PANSS of -9.7 at week 4) was low relative to that seen in other recent antipsychotic trials.

At the end of the 4-week trial, participants were given the option to enrol in an open-label study in which they received ulotaront (25-75mg) for 26 weeks. 156 patients (80.8% of those who completed the short-term trial) chose to participate in the extension study, 78 from the ulotaront group and 79 from the placebo group. Among the group who had initially been assigned to receive ulotaront and then continued treatment, the mean additional change in PANSS score over the 26 weeks from the start of the extension study was -17.1. Among the group who had initially received placebo and then switched to ulotaront in the extension study, the mean additional change in PANSS score from the start of the extension study was -27.9 (Koblan, Kent et al. 2020). Open-label extension treatment with ulotaront was also associated with a small improvement in cognitive performance (Milanovic, Origala et al. 2020).

Seven further trials are in progress to further assess ulotaront’s efficacy and safety (Table 10), and their results, due in the next couple of years, will be important given the small total number of participants in studies published so far. Two studies are phase III placebo-controlled RCTs assessing change in PANSS total score in patients experiencing an acute relapse of schizophrenia. Three trials are phase I studies investigating change in brain dopamine synthesis capacity, frequency of side effects and effect on QTc respectively. Two trials are phase III studies further investigating the incidence of side effects and serious adverse events in larger sample sizes.

### 11.3 Side effects

The adverse events that occurred during the 4-week randomised controlled trial at a frequency of at least 2% and which were more common in the ulotaront group than placebo were somnolence (6.7%), agitation (5%), nausea (5%), diarrhoea (2.5%) and dyspepsia (2.5%). The incidence of extrapyramidal symptoms was low; 3.3% in the ulotaront group and 3.2% in the placebo group (Koblan, Kent et al. 2020).

There were two serious adverse events in the ulotaront group. One of these was worsening of schizophrenia, and the second was sudden cardiac death in a patient with a history of hypertension. This patient was found to have coronary artery disease and pulmonary embolism on autopsy. There were four serious adverse events in the placebo group; three patients had worsening of schizophrenia and one attempted suicide. There was no suicidal ideation or behaviour in the ulotaront group (Koblan, Kent et al. 2020).

In the 26-week extension study, the most common adverse events were exacerbation of schizophrenia (12.2%), headache (11.5%), insomnia (8.3%), anxiety (5.1%) and somnolence (4.5%). The incidence of extra-pyramidal symptoms was similar to that in the short-term study at 3.2%. 15 of 156 patients experienced a serious adverse event; exacerbation of schizophrenia (n=11), exacerbation of psychotic disorder (n=1), acute psychosis (n=1), suicidal ideation (n=1), uterine haemorrhage (n=1) and depression (n=1). There were no deaths. Three patients reported suicidal ideation and one made an aborted attempt ((Koblan, Kent et al. 2020), supplementary appendix).

There was no clinically significant increase in metabolic laboratory values or in prolactin with ulotaront. The mean change in weight in the SEP363856 group was +0.3kg after 4 weeks and - 0.32kg after 26 weeks. There was no prolongation of corrected QT interval in the short term study; in the extension study one patient showed a≥ 60msec increase in corrected QT, although no patient had a corrected QT ≥480 msec ((Koblan, Kent et al. 2020), supplementary appendix).

### 11.4 Summary

Ulotaront is a TAAR1 and 5HT1A agonist with a low affinity for D2 receptors. It potentially acts on the dopaminergic pathophysiology of schizophrenia by inhibiting dopaminergic neurons and reducing striatal dopamine synthesis capacity. The results of the one RCT to date showed greater improvement in the ulotaront group relative to placebo over a 4-week treatment period. One maintenance study indicated that ulotaront resulted in a sustained improvement in symptoms in patients with schizophrenia, but further long and short term studies are needed to better determine the efficacy of ulotaront as well as to investigate its efficacy compared to existing antipsychotics.

Ulotaront does not appear to have a significant incidence of extrapyramidal symptoms or metabolic effects relative to placebo. The main side-effects in excess of placebo are sedation or agitation, although with a low incidence (less than 10% for each). Further safety testing in larger patient cohorts is needed to establish the incidence of rare adverse events.

## 12 Xanomeline

### 12.1 Pharmacology

Xanomeline is a muscarinic agonist with selectivity for M1 and M4 receptor subtypes (K_i_= 79.4 nM and 20.0 nM respectively, Table 1) (Shekhar, Potter et al. 2008, Watson, Brough et al. 1998). It has the highest affinity for muscarinic receptors of novel antipsychotics discussed here (Figure 3). Some established antipsychotics including chlorpromazine, olanzapine and clozapine also bind to muscarinic receptors with similar affinity to xanomeline, but they predominantly act as antagonists at muscarinic receptors (Table 1), in contrast to xanomeline. Various evidence points to the involvement of the muscarinic cholinergic system in schizophrenia, in particular lower levels of muscarinic receptors have been found in patients with schizophrenia both post mortem and in in vivo imaging studies (McKinzie, Bymaster 2012).

Xanomeline has little affinity for dopamine receptors (K_i_ values for D2 and D3 receptors are 1000 and 398.1 nM respectively, Table 1), but despite this xanomeline demonstrates functional dopamine antagonism in rodent models (Shekhar, Potter et al. 2008) and has been shown to inhibit dopamine cell firing in the limbic ventral tegmental area in rodent cell recordings (Bymaster, Shannon et al. 1998). Some work has found xanomeline to have some affinity for serotonergic receptors (K_i_ values for 5HT1a, 5HT2a and 5HT2c receptors being 63.1, 125.9 and 39.8 nM respectively) (Roth, Lopez, Watson, Brough et al. 1998), though this was not replicated by other research groups (Felder, Bymaster et al. 2000).

There is significant first-pass metabolism of xanomeline, resulting in low oral bioavailability of <1% (Mirza, Peters et al. 2003). Following oral administration, peak plasma levels are reached after 2.5 hours (Mirza, Peters et al. 2003). Xanomeline has a half-life of 4.56 hours, with steady state thus attained 23 hours after oral administration (Bymaster, Whitesitt et al. 1997).

### 12.2 Clinical efficacy

Table 11 shows the two clinical trials of xanomeline in schizophrenia to date. A randomised placebo-controlled trial of xanomeline in 20 subjects with schizophrenia or schizoaffective disorder found that the xanomeline group had significantly better outcomes for total PANSS score, total BPRS score and some cognitive tests (though the statistical tests for cognition were not adjusted for multiple testing) (Shekhar, Potter et al. 2008). All subjects in this trial received placebo for the first week of the study, which may have unblinded the treatment arm if they noticed a difference in the second week of the study when they were switched from placebo to xanomeline.

A more robust, recent RCT tested the combination of xanomeline and trospium in 182 patients experiencing an acute relapse of schizophrenia over 5 weeks (Brannan, Sawchak et al. 2021). Trospium is a peripherally acting muscarinic receptor antagonist which does not cross the blood brain barrier (Rovner 2004). Therefore, the coformulation of xanomeline and trospium is hoped to result in therapeutic efficacy without peripheral cholinergic adverse effects (Brannan, Sawchak et al. 2021). Patients in the xanomeline-trospium group had significantly greater reductions in PANSS total, PANSS positive subscale and PANSS negative subscale scores compared to placebo (Brannan, Sawchak et al. 2021).

### 12.3 Side-effect profile

The two clinical trials of xanomeline in schizophrenia detailed above are also the only two trials of its tolerability in patients with schizophrenia. Xanomeline’s action on muscarinic receptors gives it a potential for causing a number of peripheral side effects. In the Shekhar et al trial, nausea (70%), vomiting (60%), gastrointestinal distress (70%), salivation (20%), diarrhoea (20%) and constipation (20%) were all more common in the xanomeline group compared to placebo, though this study had a small sample size of 20 (Shekhar, Potter et al. 2008).

Similarly, in their larger study of 182 subjects treated with a xanomeline-trospium combination, Brannan et al found the most common adverse events in the xanomeline-trospium group were constipation (17%), nausea (17%), dry mouth (9%) and vomiting (9%) (Brannan, Sawchak et al. 2021). This suggests that, although the addition of trospium reduces gastrointestinal effects of xanomeline, it does not abolish them. Rates of treatment discontinuation for the xanomeline-trospium combination were similar in the active treatment and control groups (20 and 21% respectively) (Brannan, Sawchak et al. 2021).

There was no significant increase in weight in the Brannan et al study for patients on xanomeline-trospium relative to placebo. While subjects in the treatment group had a peak mean increase in heart of 6.9 beats per minute at day 8 (compared to +1.4 beats per minute for the placebo group), there was no significant between-group difference in blood pressure or corrected QT. There were also similar rates of EPSEs and akathisia for the treatment and placebo groups. There were no significant differences in the incidences of sedation or agitation with xanomeline-trospium compared to placebo (Brannan, Sawchak et al. 2021). Neither the Shekhar et al nor the Brannan et al studies, nor any other trials have investigated the effect of xanomeline on serum prolactin to date.

In the Brannan et al study, one serious and one severe adverse event occurred in the xanomeline-trospium group, and one severe adverse event occurred in the treatment group. However, no further detail was given as to the nature of these (Brannan, Sawchak et al. 2021).

### 12.4 Summary

Xanomeline acts as a muscarinic agonist but also exhibits functional dopamine antagonism, particularly in the ventral tegmental area. An initial small RCT and a subsequent much larger RCT have both found it to be effective in alleviating positive, negative and cognitive symptoms of schizophrenia compared to placebo, though statistical tests for cognitive symptoms were not adjusted for multiple testing. While xanomeline is not associated with those adverse effects related to conventional antipsychotics such as extrapyramidal and metabolic effects, gastrointestinal side effects were relatively common. Longer term studies are needed to assess the effectiveness of xanomeline in preventing relapse, as well as further tolerability studies to determine the incidence and nature of severe adverse events.

## 13 BI 409306

### 13.1 Pharmacology

BI 409306 is a phosphodiesterase 9A (PDE9A) inhibitor developed to target cognitive impairment in schizophrenia and Alzheimer’s disease (Brown, Daniels et al. 2018). PDE9A hydrolyses cyclic guanosine monophosphate (cGMP) and regulates its intracellular concentration within glutamatergic neurons (Dorner-Ciossek, Kroker et al. 2017). PDE9A inhibition may thus increase intracellular cGMP availability and increase NMDA receptor signalling to enhance synaptic plasticity and memory function (Brown, Daniels et al. 2018).

BI 409306 is rapidly absorbed and eliminated. Maximum plasma concentration is reached 30-45 minutes after oral administration and its elimination half-life is 1.10 – 1.85 hours (Brown, Daniels et al. 2018).

### 13.2 Clinical Efficacy

There has been one phase II study of BI 409306 which did not find a significant effect of adjunctive BI 409306 on the primary outcome measure of change in MCCB (MATRICS (Measurement and Treatment Research to Improve Cognition in Schizophrenia) Consensus Cognitive Battery) score over placebo (Table 12) (Brown, Nakagome et al. 2019). There was also no significant difference between the treatment and placebo arms for the secondary endpoints of Schizophrenia Cognition Rating Scale score or Clinical Global Impressions- Severity scale score (Brown, Nakagome et al. 2019). Two further clinical trials of BI 409306 (NCT03351244 and NCT03230097) were in progress but were terminated due to the covid-19 pandemic.

### 13.3 Side effect profile

PDE9A is expressed in the inner retina where cGMP regulates signalling in retinal cells (Dhingra, Tummala et al. 2014). Visual symptoms have been reported in safety studies of BI 409306 in healthy volunteers (Moschetti, Boland et al. 2016, Boland, Moschetti et al. 2017). In the phase II study in subjects with schizophrenia, eye disorders (blurred vision, photophobia, visual brightness, flashes, colour disturbance) occurred in 11.1% of participants receiving BI 409306, with a dose-dependent relationship in frequency (Brown, Nakagome et al. 2019). Other adverse events that occurred at higher rates in the treatment group with a frequency of ≥ 2% were nasopharyngitis (3.2%), nausea (2.6%) and dizziness (2.6%). No patients reported extrapyramidal side effects and rates of insomnia were similar to placebo (1.7% with BI 409306 vs 1.2% with placebo). 1.5% of participants receiving BI 409306 experienced a ‘cardiac disorder’ (compared to 1.5% of the placebo group), though none of these were classed as severe. There was no data regarding effect of BI 409306 on QTc. There were no serious adverse events in the treatment group (Brown, Nakagome et al. 2019).

### 13.4 Summary

BI409306 is a phosphodiesterase 9A inhibitor which has so far been tested in one short-term phase II study with no significant benefit in cognitive outcomes over placebo. Visual symptoms make up the most common reported side effects.

## 14 BI 425809

### 14.1 Pharmacology

Like BI409306, BI 425809 has also been developed to target cognition and memory in schizophrenia and Alzheimer’s disease (Moschetti, Schlecker et al. 2018). BI 425809 is a glycine transporter 1 (GlyT1) inhibitor that aims to target glutamatergic pathways, by increasing synaptic glycine levels to augment glutamate’s action at NMDA receptors (Moschetti, Schlecker et al. 2018).

BI 425809 reaches maximum plasma concentration between 3 and 4.5 hours after oral administration, and steady-state is reached after 6-10 days. It has a long half-life of over 30 hours (Moschetti, Schlecker et al. 2018).

### 14.2 Clinical efficacy

There was a statistically significant improvement on the MCCB (Measurement and Treatment Research to Improve Cognition Consensus Cognitive Battery) overall composite T-score in the BI 425809 10mg and 25mg groups of NCT02832037, though the effect sizes were small-moderate (0.34 and 0.30 for 10mg and 25mg respectively) so the clinical significance remains unclear (Table 13) (Fleischhacker, Podhorna et al. 2021). In addition, there was no statistically significant improvement in secondary outcome measures assessing social and daily functioning (Fleischhacker, Podhorna et al. 2021).

A further four clinical trials are in progress. Three phase III trials (NCT04846868, NCT04846881 and NCT04860830) will provide information regarding effect on cognitive symptoms over a longer follow-up period of 26 weeks, and one phase II trial (NCT03859973) will assess the value of combining BI 425809 and adjunctive computerised cognitive training efficacy.

### 14.3 Side effect profile

The most frequent adverse events reported in NCT02832037 which were more frequent for the BI 425809 treatment groups than placebo were headache (8-12% incidence for BI 425809), somnolence (2-6%) and gastrointestinal symptoms (2-11%) (Fleischhacker, Podhorna et al. 2021). Overall in the study, BI 425809 was associated with a dose-dependent decrease from baseline in haemoglobin levels and anaemia occurred in 1-5% of treatment groups. This is an expected class effect of GlyT1 inhibitors that has been reported with other glycine transporter inhibitors (Fleischhacker, Podhorna et al. 2021, Bugarski-Kirola, Dragana, Iwata et al. 2016, Bugarski-Kirola, D., Wang et al. 2014).There were no clinically relevant changes in other laboratory parameters, ocular parameters or ECG parameters. The frequency of extrapyramidal side effects was not reported, though this is not an expected adverse effect for GlyT1 inhibitors. There was no worsening of underlying disease or suicidality in the treatment arms. 3.5% of subjects receiving BI 425809 experienced a serious adverse event, compared to 2% of the placebo group. The nature of the serious adverse events were not specified further, though there were no deaths during this study (Fleischhacker, Podhorna et al. 2021).

### 14.4 Summary

BI 425809 is a glycine transporter 1 inhibitor increasing glutamatergic transmission. One short-term phase II study found a statistically significant improvement in cognitive outcomes with the BI 425809 10mg and 25mg over placebo, though with small effect sizes. Trials in progress will assess its efficacy over a longer follow up period and determine whether its combination with computerised cognitive training could be beneficial. Glycine transporter 1 inhibitors are associated with decrease in haemoglobin. BI 425809 appears to have a favourable metabolic profile, though longer-term data is needed.

## 15 MK-8189

### 15.1 Pharmacology

MK-8189 is a phosphodiesterase 10A inhibitor which modulates both dopamine D1-direct and D2-indirect striatal pathways and regulates striatal glutamate receptor phosphorylation (Krogmann, Peters et al. 2019, Grauer, Pulito et al. 2009). It therefore targets both dopaminergic and glutamatergic dysfunction thought to underlie schizophrenia.

Following oral administration, peak plasma concentration is reached between 12 and 24 hours according to data from NCT 03565068 reported on clinicaltrials.gov (not yet published) (NIH U.S. National Library of Medicine 2021).The elimination half-life of MK-8189 is between 7.6 and 10.9 hours (NIH U.S. National Library of Medicine 2021).

### 15.2 Clinical efficacy

There has been one clinical trial assessing clinical efficacy of 12mg MK-8189 in schizophrenia thus far (Table 14). This was conducted in patients experiencing an acute relapse and gave treatment over a 4-week period (NIH U.S. National Library of Medicine 2018). MK-8189 12mg did not show a significant difference in mean PANSS total score change relative to placebo in this relatively short study, although the active control, risperidone, did separate from placebo (NIH U.S. National Library of Medicine 2018).

A further larger phase II trial is currently underway which will test MK-8189 at higher doses of 16mg and 24mg.

### 15.3 Side effect profile

The results of study NCT03565068 assessing safety and tolerability of MK-8189 have not yet been published (NIH U.S. National Library of Medicine 2021).

### 15.4 Summary

MK-8189 is a phosphodiesterase 10A inhibitor acting on dopaminergic and glutamatergic dysfunction in schizophrenia. One short-term phase II trial did not find a significant benefit from MK-8189 12mg over placebo, though a larger study is underway testing higher doses of MK-8189. Tolerability data have not yet been published.

## 16 Other compounds in development

Ralmitaront (RO6889450) acts on the TAAR1 receptor, like SEP-363856, but as a partial agonist (Gomes, Grace 2021). Two phase II studies are currently in progress to assess the efficacy of ralmitaront on acute relapse of schizophrenia and on negative symptoms (NCT03669640 and NCT04512066) (NIH U.S. National Library of Medicine 2021c, NIH U.S. National Library of Medicine 2021e).

BIIB-104 is an α-amino-3-hydroxy-5-methyl-4-isoxazole propionic acid (AMPA) receptor positive allosteric modulator currently being tested in a phase II study of cognitive impairment in schizophrenia (NCT03745820) (NIH U.S. National Library of Medicine 2021d, Kadriu, Musazzi et al. 2021). The AMPA receptor is a non-NMDA type glutamate receptor thought to be involved in synaptic plasticity inherent in learning and memory (Kadriu, Musazzi et al. 2021).

Evenamide (NW-3509) is a selective voltage-gated sodium channel blocker which inhibits synaptic release of glutamate and reduces neuronal hyperexcitability in the prefrontal cortex and hippocampus (Bhatia, Goyal et al. 2019, Singh, Sharma et al. 2019). It does not interact with dopaminergic, noradrenergic, serotonergic or histaminergic neurotransmitter systems (Singh, Sharma et al. 2019). A phase II RCT found that add-on evenamide was associated with statistically significant efficacy and good tolerability (Anand, Forrest et al. 2018, Anand, Hartman et al. 2017), though full results have yet to be published.

TAK-041 is a G-protein-coupled receptor 139 (GPCR139) agonist (Reichard, Schiffer et al. 2021). GPCR 139 is highly expressed in the habenula, a brain nucleus involved in transducing information from the forebrain to dopaminergic and serotonergic systems in the midbrain and brainstem and thought to be implicated in schizophrenia (Reichard, Schiffer et al. 2021). A phase II RCT of add-on TAK-041 to existing antipsychotic treatment in 23 subjects did not find a statistically significant improvement in cognitive function with TAK-041 according to the summary online (NCT03319953) (NIH U.S. National Library of Medicine 2021b), although, as the full study report has not been published to date, it is not clear if it was powered to detect a significant difference or if further studies are planned.

ALKS-3831 was developed as a combination of olanzapine with samidorphan (an opioid antagonist) (Krogmann, Peters et al. 2019). Samidorphan binds with high affinity to μ-, κ- and δ-opioid receptors and so reduces olanzapine-induced food craving by blocking receptors in the brain reward pathway (Krogmann, Peters et al. 2019). Short-term phase II and phase III studies have shown that ALKS-3831 has similar efficacy to olanzapine alone (Martin, Correll et al. 2019, Potkin, Kunovac et al. 2020). In 12-week and 6-month studies, ALKS-3831 led to significantly less weight gain, though there was no significant difference in serum glucose and lipids relative to olanzapine (Martin, Correll et al. 2019, Krogmann, Peters et al. 2019).

## 17 Conclusions

Established antipsychotics have a number of limitations in the treatment of schizophrenia including treatment resistance in up to a third of patients, limited efficacy for negative and cognitive symptoms, and poor tolerability in many patients. Our review has identified a number of pharmacological and clinical features of new and emerging drugs that could address these issues. Key features of these drugs are reviewed in the summary table (Table 15), together with our evaluation of the strength of the corresponding evidence based on the GRADE criteria. A limitation of our approach is that we have not reviewed all drugs in development for schizophrenia. However, it is not possible to review all drugs at any stage of development. Instead, our review focuses on drugs that have recently been licensed, or are in late phase development (with at least one phase II trial on clinicaltrials.gov) to provide an update on new and emerging treatments with a range of different mechanisms. It should be recognised that there are other promising compounds in development. Nevertheless, our review has identified a number of novel pharmacological approaches to treating schizophrenia with promising clinical support. In addition, we have searched company websites and other data sources to provide additional data to complement the findings on these compounds published in scientific journals.

We identified only one drug (Lu AF35700) that has been tried in treatment resistance recently, despite the major clinical and health economic burden this represents (Kane, Agid et al. 2019, Potkin, Kane et al. 2020). Whilst Lu AF35700 did not show a benefit over olanzapine/risperidone in treatment resistance, several studies with other drugs are ongoing for this indication, notably with lumateperone and pimavanserin. Of note, long term use of established antipsychotics may contribute to late-onset treatment resistance due to D2 and D3 receptor upregulation and subsequent supersensitivity to dopamine (Potkin, Kane et al. 2020). Dopamine partial agonists (cariprazine, brexpiprazole, brilaroxazine and F17464), and indeed the other novel agents with low D2 binding, are not expected to induce D2 receptor supersensitivity and so may be associated with a lower risk of inducing secondary treatment resistance (Murray, R. M., Quattrone et al. 2016). This may be a potential benefit of these novel approaches, although it needs testing in clinical populations.

For negative symptoms, emerging evidence suggests that some of the novel agents could show promise in targeting these domains. Of these drugs, only cariprazine, pimavanserin and roluperidone have been tested in studies where the primary aim was to test efficacy for negative symptoms, and cariprazine is the only drug to date that has been tested against an established antipsychotic. Whilst cariprazine showed a benefit for negative symptoms over the established antipsychotic, confirmation of this finding is required from further large prospective studies to determine if the promising initial results are robust and clinically meaningful.

With regard to cognitive symptoms, one RCT showed high-dose roluperidone was associated with an improvement in cognition over placebo, as did another RCT testing xanomeline-trospium, though cognitive tests for the latter drug were not adjusted for multiple testing. Open-label maintenance treatment of SEP-363856 was also associated with a small improvement in cognitive performance.

BI 425809 was associated with statistically significant improvement in cognition over placebo in one RCT, but BI 409306 was not. The other novel agents have not been specifically investigated for their effects on cognition to date. Lumateperone may additionally result in improvement in depressive symptoms, although this was based on a very small sample. Nevertheless, this potential benefit of lumateperone could be very useful given the high prevalence of depressive symptoms in schizophrenia, highlighting the value of further testing.

Further work is also needed to investigate the efficacy of these new treatment options relative to well-established antipsychotics and study their long-term effectiveness preventing relapse. Preliminary findings suggest cariprazine is safe in adolescent and elderly patients, but the novel agents will all require further investigation in paediatric and elderly patient cohorts before their use is expanded to these age groups. Additional trials are currently planned to test the use of cariprazine, brexpiprazole and lumateperone in children and adolescents.

With respect to side effects, all the agents we have reviewed are notable in not being high-affinity D2 antagonists, which means that the risk of EPSE and hyperprolactinemia should be lower than that seen with established antipsychotics. Lumateperone, pimavanserin, roluperidone, SEP-363856, xanomeline, BI 409306 and F17464 appear to be particularly favourable with rates of extra-pyramidal side effects very similar to placebo. In contrast, approximately 10% of patients taking cariprazine and 6% of patients on brilaroxazine experience EPSEs, although this is still lower than rates with many established drugs (Misdrahi, Tessier et al. 2019). The incidence of extra-pyramidal side effects in BI 425809, Lu AF35700 and MK-8189 has not been reported. Prolactin elevation, and its effects on sexual function and bone mineral density, is a common side effect of established antipsychotics (Howes, Oliver D., Wheeler et al. 2005, Howes, O. 2007). F17464 was the only antipsychotic among these novel agents associated with hyperprolactinaemia. Cariprazine, brexpiprazole, lumateperone, pimavanserin, roluperidone, SEP-363856 and brilarozazine did not lead to elevated prolactin levels. There is no data yet regarding the effects of xanomeline, BI 409306, BI 425809, LuAF AF35700 and MK-8189 on serum prolactin.

Metabolic and cardiovascular side-effects are a considerable concern with many established antipsychotics, particularly second generation drugs such as clozapine and olanzapine, and given that schizophrenia may be associated with an increased risk of cardiometabolic dysregulation (Pillinger, Beck et al. 2017, Osimo, Brugger et al. 2020). Pimavanserin and roluperidone have been found to induce QTc prolongation, but neither has major effects on metabolic parameters. Cariprazine, brexpiprazole, lumateperone, SEP-363856, xanomeline, BI 425809 and brilaroxazine result in little derangement of metabolic parameters or QTc interval, suggesting these could be significant advantages over established antipsychotics. F17464 was associated with hyperlipidaemia, though not with change in ECG parameters. More data is needed regarding the cardiometabolic effects of BI 409306, Lu AF35700 and MK-8189.

Lumateperone appears to have the highest propensity to cause sedation, with this affecting a quarter of patients in short term trials, corresponding to the drug’s high affinity for 5-HT2A receptors. Somnolence was also more common in patients taking roluperidone and BI 425809 over placebo. On the other hand, cariprazine, brexpiprazole, brilaroxazine and F17464 are activating with higher rates of akathisia and restlessness than sedation, in keeping with their mechanism as partial dopamine agonists (Kaar, Natesan et al. 2020). SEP-363856 and Lu AF35700 resulted in low rates of somnolence or agitation (each affecting less than 10% of patients), and for xanomeline and BI 409306 there was no evidence of increased rates of sedation or agitation relative to placebo. The frequency of sedation and agitation with MK-8189 was not specified. It, of course, remains possible that other side effects of these novel agents may emerge with larger trials and in post-marketing experience but, notwithstanding this, their favourable profiles to date represent a significant advantage over established antipsychotics.

Finally, it is important to note the varying mechanisms of action of these novel agents. While cariprazine, brexpiprazole, lumateperone, brilaroxazine and F17464 act directly on dopamine receptors, the discoveries that pimavanserin, roluperidone, SEP-363856 and xanomeline reduce positive symptoms without acting directly on D2 receptors are important potential demonstrations that antipsychotic efficacy can be independent of direct action at D2/3 receptors. If these findings are confirmed in phase III trials in schizophrenia, this would represent a key advancement in our understanding of the pathophysiology of psychosis and opens up new avenues for future drug development based on non-D2 blocking approaches.

## Figures and Tables

**Table 1:** Mean Ki values of new and established antipsychotics showing varying affinities to dopamine, serotonin, alpha-adrenergic, histamine and muscarinic receptors All values in nM

	D1	D2	D3	D4	5HT1A	5HT2A	5HT2C	α1	α2	H1	M1	M3	M4
Haloperidol^1^	83	1.4	2.9	3	1808	84.7	5000	4.7	1202.3	851.1	4374	1000	>10000
Chlorpromazine^1^	112	2.3	2.4	15.7	3057.5	2.8	15.6	1.4	281.8	2.8	39.8	57	77
Amisulpride^1^	10001	2.2	2.4	2369	10000	8304	10000	7100	1600	10000	>10000	10000	>10000
Risperidone^1^	267	2.2	6	7.8	420	0.4	23.5	1.4	5.1	18.8	7035	10000	7633
Olanzapine^1^	52.5	20.4	43.8	28.3	2063	3.2	12.1	55	173.8	2.4	24.2	78	206
Quetiapine^1^	741.3	212.5	340	2100	320	200	1406.3	15	1000	8.7	241.8	1631.5	475.7
Aripiprazole^1^	387	1.4	1	216.5	5.6	8.7	18.7	27.6	72.4	29	6774	4677	1521
Clozapine^1^	192.5	119	242	29.5	140	6.5	15.2	4	33.1	1.8	8.71	25	17.7
Cariprazine^1,2^	No data	0.59^a^	0.085	No data	3	19	134	No data	No data	23	>1000	>1000	>1000
Brexpiprazole^1,3,4^	164	0.3^b^	1.1	6.3	0.12	0.47	34	1.99^c^	0.59^d^	19	>1000	No data	No data
Brilaroxazine^5^	No data	0.37	3.7	6	1.5	2.5	39	No data	No data	No data	No data	No data	No data
Lumateperone^6,7,8^	52	32^e^	No data	No data	No data	0.54	173	73^e^	<100	>1000	>100	>100	>100
F17464^9-11^	>10 000	9.3^f^	0.17	>10 000	0.16	436.5	1995	794.3^g^	255.4^h^	21.4	>10 000	>10 000	No data
Lu AF35700	No data	No data	No data	No data	No data	No data	No data	No data	No data	No data	No data	No data	No data
Pimavanserin^12,13^	>300	>300	>300	No data	No data	0.087	0.44	>300	>300	>300	>300	>300	>300
Roluperidone^14-16^	No data^i^	No data^i^	No data^i^	No data^i^	No data	7.53^j^	No data	14.43^j^	No data	No data	No data	No data	No data
Ulotaront^17^	No data	21300	No data	No data	284	17250	2450	No data	1245^k^	No data	No data	No data	No data
Xanomeline^1^	No data	1000	398.1	No data	63.1	125.9	39.8	1584^l^	No data	No data	79.4	39.8	20.0
BI 409306	No data	No data	No data	No data	No data	No data	No data	No data	No data	No data	No data	No data	No data
BI 425809	No data	No data	No data	No data	No data	No data	No data	No data	No data	No data	No data	No data	No data
MK-8189	No data	No data	No data	No data	No data	No data	No data	No data	No data	No data	No data	No data	No data

Footnotes a: D2 long=0.49nM, D2 short=0.69nM b: D2 long=0.3nM, no data for D2 short c: α1a =3.8nM, α1b =0.17nM d: α2c =0.59nM, no data for other α2 receptor subtypes e: data from rat receptors f: D2 long=12.1nM, D2 short=6.6nM g: α1b=794.3nM, no data for other α1 receptor subtypes h: α2a=281.8nM, α2b=208.9nM, α2c=275.4nM i: IC50 > 1000nM at dopamine receptors j: not reported whether data from human brain tissue k: α2a =590nM, α2b =1900nM l: α1b= 1584 nM

Sources: 1: Roth, Lopez 2: Citrome 2013 3: Stahl 2017 4: Nerkar, Bhise 5: Cantillon, Prakash et al. 2017 6: Snyder, Vanover et al. 2015 7: Meyer 2020 8: Blair 2020 9: Bitter, I., Groc et al. 2017 10: Cosi, N’Guyen et al. 2017 11: Cosi, Martel et al. 2021 12: ACADIA Pharmaceuticals Inc 2016 13: Vanover, Weiner et al. 2006 14: Davidson, Saoud et al. 2017 15: Luthringer 2016 16: Luthringer, Pellegrini et al. 2013 17: Dedic, Jones et al. 2019

**Table 2:** Clinical efficacy trials of cariprazine in schizophrenia

Identifier	Reference	Cariprazine dose(s)	Comparator group(s)	Indication	Patient group	Type of study, phase	Primary outcome measure	Number of patients enrolled	Length of treatment	Status	Location	Year	Results	
													Change in cariprazine group relative to comparator group	Effect size
NCT04578756		Cariprazine (dose not stated)	No comparator group	SCZ and bipolar	Children and adolescents 10-18 years	Open label flexible dose study, phase III	Incidence of AEs	200 planned	26 weeks	Planned	USA	2020 – 2023 (est.)	Results due 2023	
NCT03817502		Cariprazine 1.5mg, 4.5mg	Placebo	SCZ	Adolescents 13-17 years	Double blinded RCT, phase III	Change from baseline in PANSS total score	330 planned	6 weeks	In progress	USA, Russia & Eastern Europe	2019 – 2022 (est.)	Results due 2022	
NCT03593213		Cariprazine 3mg, 4.5mg	Placebo	SCZ	PANSS ≥70 and ≤ 120	Double blinded RCT, phase III	Time from baseline to first relapse date	572	30 weeks	Terminated	USA, Asia, Eastern Europe & Central America	2018-2021	Study terminated: FDA released drug company from its post-marketing requirement	
	(Smulevich, Ivanov et al. 2020)	Cariprazine 1.5 – 6mg	No comparator group	SCZ	Predominant negative symptoms	Observational open-label study	Change in PANSS-NS and CAINS	60	4 weeks	Completed	Russia	2020	N/A	Mean change from baseline -4.3 in PANSS-NS (p<0.05) and -4.9 in CAINS (p<0.05)
	(Rancans, Dombi et al. 2021)	Cariprazine 1.5 - 6mg	No comparator group	SCZ	Outpatients with negative symptoms	Observational open label study	Change in SAND	116	16 weeks	Completed	Latvia	2018-2020	N/A	Mean change from baseline -7.3 (p<0.001) over 16 weeks
	(Németh, Laszlovszky et al. 2017)	Cariprazine 3mg, 4.5mg or 6mg	Risperidone 3mg, 4mg or 6mg	SCZ with persistent negative symptoms	Chronic, stable SCZ	Double blinded RCT, phase III	Change in PANSS-factor score for negative symptoms	461	26 weeks	Completed	Europe & Russia	2016	↑	Cariprazine vs risperidone: Mean difference -1.46, p=0.002, effect size 0.31
NCT01412060	(Durgam, Earley et al. 2016)	Cariprazine 3, 6 and 9mg	Placebo	SCZ	PANSS ≥ 70	Open label-phase (20 weeks) followed by randomised parallel-group study, phase III	Time to the first symptom relapse	765 in open label phase, 200 in double-blind phase	72 weeks	Completed	USA, India, & Eastern Europe	2011-2014	↑	Cariprazine 224 days, placebo 92 days Hazard ratio 0.45, p=0.001 Relapse in 47.5% of placebo group vs 24.8% of cariprazine group
NCT00404573	(Durgam, Litman et al. 2016)	Cariprazine 1.5-4.5mg, cariprazine 6-12mg,	Placebo	SCZ	Acute relapse	Double blinded RCT, phase III	Change in PANSS total score	392	6 weeks	Completed	USA	2006-2007	↔	No significant differences between comparator groups after correction for multiple comparisons.
NCT01104792	(Nasrallah, Earley et al. 2017)	Cariprazine 3mg, 4.5mg, 6mg, 9mg	No comparator group	SCZ	Stable schizophrenia, diagnosis > 1 year	Open label study, phase III	Change in PANSS total score	752	48 weeks	Completed	USA, South America, Eastern Europe & Asia	2010 - 2013	N/A	Mean change from baseline -5.0 over 48 weeks
NCT01104766	(Durgam, Cutler et al. 2015)	Cariprazine 3mg, cariprazine 6mg	Aripiprazole 10mg, placebo	SCZ	Acute relapse	Double blinded RCT, phase III	Change in PANSS total score	617	6 weeks	Completed	USA, Russia & Eastern Europe	2010 - 2011	↑ vs placebo ↔ vs aripiprazole	Cariprazine 3mg vs placebo: mean difference -6, p=0.004 Cariprazine 6mg vs placebo: mean difference -8.8, p<0.001 Aripiprazole 10mg/day vs placebo: mean difference -7.0, p<0.001
NCT01104779	(Kane, Zukin et al. 2015)	Cariprazine 3-6mg, cariprazine 6-9mg	Placebo	SCZ	Acute relapse	Double blinded RCT, phase III	Change in PANSS total score	446	6 weeks	Completed	USA, South America, Asia & Africa	2010 - 2011	↑	Cariprazine 3-6mg vs placebo: mean difference -6.8, p=0.003 Cariprazine 6-9mg vs placebo: mean difference -9.9, p<0.001.
NCT00694707	(Durgam, Starace et al. 2014)	Cariprazine 1.5mg, cariprazine 3mg, cariprazine 4.5mg	Risperidone 4mg, placebo	SCZ	Acute relapse	Double blinded RCT, phase II	Change in PANSS total score	732	6 weeks	Completed	USA, Asia & Eastern Europe	2008 - 2009	↑ vs placebo	Cariprazine 1.5mg vs placebo: mean difference -7.6, p<0.001 Cariprazine 3mg vs placebo: mean difference -8.8, p<0.001 Cariprazine 4.5mg vs placebo: mean difference -10.4, p<0.001 Risperidone vs placebo: mean difference -15.1, p<0.001

**Key to abbreviations and symbols:** SCZ: Schizophrenia RCT: Randomised controlled trial AE: Adverse events PANSS: Positive and negative symptom scale FDA: United States Food and Drug Administration SAND: Short Assessment of Negative Domains PANSS-NS: Positive and negative symptom scale, Negative subscale CAINS: Clinical Assessment Interview for Negative Symptoms ↑ : Better outcome in cariprazine group relative to comparator group (statistically significant) ↓ : Poorer outcome in cariprazine group relative to comparator group (statistically significant) ↔ : No statistically significant difference between cariprazine and comparator groups

**Table 3:** Clinical efficacy trials of brexpiprazole in schizophrenia

Identifier	Reference	Brexpiprazole dose(s)	Comparator group (s)	Indication	Patient group	Type of study, phase	Primary outcome measure	Number of patients enrolled	Length of treatment	Status	Location	Year	Results	
													Change in brexpiprazole group relative to comparator group	Effect size
NCT04641780		Brexpiprazole 2 – 4mg (for SCZ)	No comparator group	SCZ MDD		Prospective cohort study, phase III	Incidence of AEs	300 planned	8 weeks	Recruiting	Philippines	2019 – 2024 (est)	Due 2024	
NCT03238326		Brexpiprazole 1-4mg	No comparator group	SCZ	Adolescents aged 13 - 17	Open label study, phase III	Frequency and severity of AEs	350 planned	Up to 24 months	Recruiting	USA	2017 – 2023 (est)	Due 2023	
NCT03526354		Brexpiprazole 4mg	Treatment as usual	Co-morbid SCZ and and substance misuse disorder	Psychiatrically stable	Open label RCT, phase IV	Number of days of substance use, change in visual analogue scale measure for craving	80 planned	12 weeks	Recruiting	USA	2018 – 2022 (est)	Due 2022	
NCT03874494		Brexpiprazole 2-4mg	Aripiprazole 10-20mg	SCZ	Acute relapse	Double blinded RCT, phase III	Change in PANSS total score	370 planned	6 weeks	Recruiting	China	2019 – 2021 (est)	Due 2021	
NCT03198078		Brexpiprazole 2-4mg,	Aripiprazole 10-20mg Placebo	SCZ	Adolescents 13-17 years	Double blinded RCT, phase III	Change in PANSS total score	480 planned	6 weeks	Recruiting	USA	2017 – 2021 (est)	Due 2021	
NCT01810783	(Hakala, Gislum et al. 2018)	Brexpiprazole 1-4mg	No comparator group	SCZ	Clinically stable	Open label, phase III	Frequency of AEs	210	52 weeks	Completed	USA, Eastern Europe	2013 - 2017	N/A	Mean change in PANSS total from baseline to week 52 was -6.8 (95% CI -9.3, -4.2)
NCT01397786“ZENITH trial”	(Forbes, Hobart et al. 2018)	Brexpiprazole 1-4mg	No comparator group	SCZ	Outpatients	Open label phase III	Frequency of AEs	1072	52 weeks	Completed	North America, South America, Europe, Asia	2011-2017	N/A	Mean change in PANSS total from baseline to week 52 was -12.2.
NCT01668797 “EQUATOR trial”	(Fleischhacker, Hobart et al. 2017)	Brexpiprazole 1-4mg	Placebo	SCZ	Acute relapse	Double blinded RCT, phase III	Time from randomisation to relapse	524	52 weeks	Completed	North America, South America, Asia & Eastern Europe	2012 - 2015	↑	Time to impending relapse delayed with brexpiprazole treatment compared with placebo (p<0.01). Hazard ratio of 0.292.
	(Ishigooka, Iwashita et al. 2018)	Brexpiprazole 1mg, 2mg, 4mg	Placebo	SCZ	Acute relapse	Double blinded RCT, phase II/III	Change in PANSS total score	459	6 weeks	Completed	Japan	2011-2015	↑ for brexpiprazole 2mg only	Brexpiprazole 1mg vs placebo: mean difference -0.63, p=0.83↑Brexpiprazole 2mg vs placebo: mean difference -7.32, p=0.01↑Brexpiprazole 4mg vs placebo: mean difference -3.86, p=0.20
NCT02054702	(Citrome, Ota et al. 2016)	Brexpiprazole 3mg	Aripiprazole 15mg	SCZ	Acute relapse	Randomised open label study, phase II	Change in PANSS total score	97	6 weeks	Completed	USA	2014	↔	Mean reduction in PANSS total score -22.9 for brexpiprazole (p<0.0001 vs baseline) and -19.4 (p<0.0001 vs baseline) for aripiprazole
NCT01810380 “Lighthouse trial”	(Marder, Eriksson et al. 2020)	Brexpiprazole 2-4mg	Quetiapine extended release 400-800mg, Placebo	SCZ	Acute relapse	Double blinded RCT, phase III	Change in PANSS total score	468	6 weeks	Completed	USA	2013-2014	↓	Brexpiprazole vs placebo: mean difference -4.1, p=0.056 Quetiapine vs placebo: mean difference -8.0, p<0.01
NCT02013622	(ClinicalTrials.gov 2016)	Brexpiprazole 1-4mg	No comparator group	SCZ	Early episode SCZ	Open label study, phase III	Change in PANSS total score	49	16 weeks	Completed	USA	2013-2014	N/A	Mean change in PANSS from baseline -10.2 (p<0.01).
NCT01396421“VECTOR trial”	(Correll, Skuban et al. 2015)	Brexpiprazole 0.25mg, 2mg, 4mg	Placebo	SCZ	Acute relapse	Double blinded RCT, phase III	Change in PANSS total score	623	6 weeks	Completed	North America, Eastern Europe & Asia	2011-2013	↑ for brexpiprazole 2mg and 4mg	Brexpiprazole 0.25mg vs placebo: mean difference -2.89, p=0.30 Brexpiprazole 2mg vs placebo: mean difference -8.72, p<0.01 Brexpiprazole 4mg vs placebo: mean difference -7.64, p<0.01
NCT01393613“BEACON trial”	(Kane, Skuban et al. 2015)	Brexpiprazole 1mg, 2mg, 4mg	Placebo	SCZ	Acute relapse	Double blinded RCT, phase III	Change in PANSS total score	674	6 weeks	Completed	USA, Central America, South America, Eastern Europe & Asia	2011 - 2014	↑ for brexpiprazole 4mg only	Brexpiprazole 1mg vs placebo: mean difference -3.37, p=0.16 Brexpiprazole 2mg vs placebo: mean difference -3.08, p=0.14 Brexpiprazole 4mg vs placebo: mean difference -6.47, p<0.01.
NCT00905307; STEP 203	(Correll, Skuban et al. 2016)	Brexpiprazole 0.25mg, 1mg, 2.5mg, 5mg	Aripiprazole 15mg Placebo	SCZ	Acute relapse	Double blinded RCT, phase II	Change in PANSS total score	459	6 weeks	Completed	USA, Asia & Europe	2009 - 2010	↔	Brexpiprazole 0.25mg vs placebo: mean difference 4.88, p=0.23 Brexpiprazole 1mg vs placebo: mean difference -4.69, p=0.13 Brexpiprazole 2.5mg vs placebo: mean difference -1.72, p=0.58 Brexpiprazole 5mg vs placebo: mean difference -4.45, p=0.15 Aripiprazole 15mg vs placebo: mean difference -3.68, p=0.31

**Key to abbreviations and symbols:** SCZ: Schizophrenia MDD: Major depressive disorder AEs: Adverse events PANSS: Positive and negative symptom scale RCT: Randomised controlled trial CI: Confidence interval ↑ : Better outcome in brexpiprazole group relative to comparator group (statistically significant) ↓ : Poorer outcome in brexpiprazole group relative to comparator group (statistically significant) ↔ : No statistically significant difference between brexpiprazole and comparator groups

**Table 4:** Clinical efficacy trials of brilaroxazine in schizophrenia

Identifier	Reference	Brilaroxazine dose(s)	Comparator group(s)	Indication	Patient group	Type of study, phase	Primary outcome measure	Number of patients enrolled	Length of treatment	Status	Location	Year	Results	
													Change in brilaroxazine group relative to comparator group	Effect size
NCT01490086	(Cantillon, Prakash et al. 2017)	Brilaroxazine 15mg, 30mg, 50mg	Aripiprazole 15mg, placebo	SCZ or SZA	Acute relapse	Double blinded RCT, phase II	Change in PANSS score	234	28 days	Completed	North America, Europe & Asia	2011-2015	↑	Brilaroxazine 15mg vs placebo: mean difference -8.82, p=0.021 Brilaroxazine 30mg vs placebo: mean difference -4.01, p=0.273 Brilaroxazine 50mg vs placebo: mean difference -7.8, p=0.016 Aripiprazole 15mg/day vs placebo: mean difference +2.09, p=0.556

**Key to abbreviations and symbols:** SCZ: Schizophrenia SZA: Schizoaffective disorder RCT: Randomised controlled trial AE: Adverse events ↑ : Better outcome in brilaroxazine group relative to comparator group (statistically significant) ↓ : Poorer outcome in brilaroxazine group relative to comparator group (statistically significant) ↔ : No statistically significant difference between brilaroxazine and comparator groups

**Table 5:** Clinical efficacy trials of lumateperone in schizophrenia

Identifier	Reference	Lumateperone dose(s)	Comparator group(s)	Indication	Patient group	Type of study, phase	Primary outcome measure	Number of patients enrolled	Length of treatment	Status	Location	Year	Results	
													Change in lumateperone group relative to comparator group	Effect size
NCT04779177		Lumateperone 42mg, 28mg	No comparator group	SCZ or SZA	Adolescents aged 13-17 years, clinically stable	Open label study, phase I	Pharmacokinetic data, frequency of AEs	12 planned	5 days	Planned	USA	March-Oct 2021 (est.)	Due October 2021	
NCT04709224		Lumateperone tosylate 50mg, 100mg, 200mg long-acting injection	No comparator group	SCZ	Clinically stable	Open label study, phase I	Pharmacokinetic data, frequency of AEs	24 planned	Single dose long-acting injection	In progress	USA	2020-2021 (est.)	Due December 2021	
NCT03817528		Lumateperone tosylate 40-60mg	N No comparator group	SCZ	Inadequate response or tolerability to previous antipsychotics	Open label study, phase II	Change in PANSS total score	40 planned	6 months	Terminated	USA	2019-2021 (est.)	Terminated 2021: Lumateperone approved by FDA	
ITI-007-303	(Correll, Davis et al. 2020)	Lumateperone 42mg (lumateperone tosylate 60mg)	No comparator group	SCZ	Clinically stable outpatients	Open label safety study, phase III	Incidence of AEs	302	6 weeks	Completed	USA	2017-2018	N/A	At day 42, mean change in PANSS from previous antipsychotic baseline was -2.2 (p<0.001)
NCT02469155; ITI-007-302	(Vanover, Dmitrienko et al. 2018)	Lumateperone tosylate 20mg, 60mg	Risperidone 4mg, Placebo	SCZ	Acute relapse	Double blinded RCT, phase III	Change in PANSS total score	696	6 weeks	Completed	USA	2015-2016	↔	Lumateperone tosylate 20mg vs placebo: mean difference 0.1, p>0.05 Lumateperone tosylate 60mg vs placebo: mean difference 0.5, p>0.05 Risperidone 4mg vs placebo: mean difference -5.4, p<0.05
NCT02282761; ITI-007-301	(Correll, Davis et al. 2020)	Lumateperone tosylate 40mg, 60mg	Placebo	SCZ	Acute relapse	Double blinded RCT, phase III	Change in PANSS total score	450	28 days	Completed	USA	2014-2015	↑ for lumateperone 60mg only	Lumateperone tosylate 40mg vs placebo: mean difference -2.6, p=0.16 Lumateperone tosylate 60mg vs placebo: mean difference -4.2, p=0.02
NCT01499563; ITI-007-005	(Lieberman, Davis et al. 2016)	Lumateperone 60mg, 120mg	Risperidone 4mg, placebo	SCZ	Acute relapse	Double blinded RCT, phase II	Change in PANSS total score	335	28 days	Completed	USA	2011-2013	↑ for lumateperone 60mg only ↔ vs risperidone	Lumateperone 60mg vs placebo: mean difference -5.8, p=0.017 Lumateperone 120mg vs placebo: mean difference -0.9, p=0.71 Risperidone 4mg vs placebo: mean difference -6.0, p=0.013

**Key to abbreviations and symbols:** SCZ: Schizophrenia SZA: Schizoaffective disorder CGI-S: Clinical Global Impression-Severity score RCT: Randomised controlled trial PANSS: Positive and negative symptom scale FDA: United States Food and Drug Administration AEs: Adverse events ↑ : Better outcome in lumateperone group relative to comparator group (statistically significant) ↓ : Poorer outcome in lumateperone group relative to comparator group (statistically significant) ↔ : No statistically significant difference between lumateperone and comparator groups

**Table 6:** Clinical efficacy trials of F17464 in schizophrenia

Identifier	Reference	F17464 dose(s)	Comparator group(s)	Indication	Patient group	Type of study, phase	Primary outcome measure	Number of patients enrolled	Length of treatment	Status	Location	Year	Results	
													Change in F17464 group relative to comparator group	Effect size
NCT02151656	(Bitter, Istvan, Lieberman et al. 2019)	F17464 20mg BD	Placebo	SCZ	Acute relapse	Double-blinded RCT, phase II	Change in PANSS total score	134	6 weeks	Completed	Europe	2014-2015	↑	F17464 40mg vs placebo: mean difference -6.2, p<0.01 [one-sided test]

**Key to abbreviations and symbols:** SCZ: Schizophrenia SZA: Schizoaffective disorder RCT: Randomised controlled trial AE: Adverse events ↑ : Better outcome in F17464 group relative to comparator group (statistically significant) ↓ : Poorer outcome in F17464 group relative to comparator group (statistically significant) ↔ : No statistically significant difference between F17464 and comparator groups

**Table 7:** Clinical efficacy trials of Lu AF35700 in schizophrenia

Identifier	Reference	Lu AF35700 dose(s)	Comparator group(s)	Indication	Patient group	Type of study, phase	Primary outcome measure	Number of patients enrolled	Length of treatment	Status	Location	Year	Results	
													Change in Lu AF35700 group relative to comparator group	Effect size
NCT03230864 (Anew)		Lu AF35700 10mg	Risperidone 4-6mg, olanzapine 15-20mg	SCZ	Treatment-resistant	Double blinded RCT, phase III	Change in PANSS score	119	8 weeks	Completed	North America, Europe, Asia	2017-2019	↔	Lu AF35700 10mg vs risperidone/olanzapine: mean difference +5.47, p=0.081
NCT02717195 (Daybreak)		Lu AF35700 10mg, 20mg	Risperidone 4-6mg, olanzapine 15-20mg	SCZ	Treatment-resistant	Double blinded RCT, phase III	Change in PANSS score	1098	10 weeks	Completed	North America, Europe	2016-2018	↔	Lu AF35700 10mg vs risperidone/olanzapine: mean difference -0.12, p=0.920 Lu AF35700 20mg vs risperidone/olanzapine: mean difference +1.67, p=0.147

**Key to abbreviations and symbols:** SCZ: Schizophrenia RCT: Randomised controlled trial PANSS: Positive and Negative Syndrome Scale AE: Adverse events ↑ : Better outcome in Lu AF35700 group relative to comparator group (statistically significant) ↓ : Poorer outcome in Lu AF35700 group relative to comparator group (statistically significant) ↔ : No statistically significant difference between Lu AF35700 and comparator groups

**Table 8:** Clinical efficacy trials of pimavanserin in schizophrenia

Identifier	Reference	Pimavanserin dose(s)	Comparator groups	Indication	Patient group	Type of study, phase	Primary outcome measure	Number of patients enrolled	Length of treatment	Status	Location	Year	Results	
													Change in pimavanserin group relative to comparator group	Effect size
NCT03121586 (ACP-103-035)	(ACADIA Pharmaceuticals Inc 2020d)	Adjunctive pimavanserin 10, 20 or 34mg + usual antipsychotic	No comparator group	SCZ	Clinically stable	Open label study, phase III	Safety and tolerability	500 planned	52 weeks	Recruiting	North America, Europe	2024 (est.)	Due March 2024	
NCT04531982		Adjunctive pimavanserin 34mg + usual antipsychotic	Placebo + usual antipsychotic	SCZ	Clinically stable	Double blinded RCT, phase III	Change in NSA-16 total score	462 planned	26 weeks	Recruiting	Europe, Russia	2020-2023 (est.)	Due March 2023	
NCT03994965	(Baltzersen, Meltzer et al. 2020)	Pimavanserin 34mg	No comparator group	SCZ spectrum^1^	Medication free, first episode psychosis	Open label study	Change in PANNS total score	40 planned	6 weeks	Recruiting	Europe	2023 (est.)	Due January 2023	
NCT02970305 (ACP-103-038)	Press release only (ACADIA Pharmaceuticals Inc 2020c, ACADIA Pharmaceuticals Inc 2019)	Adjunctive pimavanserin 10, 20 or 34mg + usual antipsychotic	Placebo and background antipsychotic	SCZ	Predominant negative symptoms	Double blinded RCT, phase II	Change in NSA-16 total score	403	26 weeks	Complete, awaiting full publication of results	North America, Eastern Europe	2016-2019	↑	Change in NSA at 26 weeks: mean difference -1.9, p=0.043
NCT02970292 (ACP-103-034) (ENHANCE-1)	Report on clinicaltrials.gov + press release (ACADIA Pharmaceuticals Inc 2020a, ACADIA Pharmaceuticals Inc 2020b)	Adjunctive pimavanserin 10, 20 or 34mg + usual antipsychotic	Placebo + usual antipsychotic	SCZ	Partial responders	Double blinded RCT, phase III	Change in PANSS total score	396	6 weeks	Complete	North America, Eastern Europe	2016-2019	↔	Change in PANSS: mean difference -1.9, p=0.094

**Key to abbreviations and symbols:** SCZ: Schizophrenia RCT: Randomised controlled trial NSA-16: Negative Symptom Assessment-16 AEs: Adverse events PANSS: Positive and negative symptom scale ^1^Schizophrenia, persistent delusional disorder, acute and transient psychotic disorders, schizoaffective disorder, other non-organic psychotic disorders and unspecified non-organic disorders ↑ : Better outcome in pimavanserin group relative to comparator group (statistically significant) ↓ : Poorer outcome in pimavanserin group relative to comparator group (statistically significant) ↔ : No statistically significant difference between pimavanserin and comparator groups

**Table 9:** Clinical efficacy trials of roluperidone in schizophrenia

Identifier	Reference	Roluperidone dose(s)	Comparator groups	Indication	Patient group	Type of study, phase	Primary outcome measure	Number of patients enrolled	Length of treatment	Status	Location	Year	Results	
													Change in roluperidone group relative to comparator group	Effect size
NCT03397134 MIN-101C07	Not published in peer-reviewed journal, preliminary results accessed from (Minerva Neurosciences Inc 2020)(Minerva Neurosciences Inc 2020)(Minerva Neurosciences Inc 2020) (Minerva Neurosciences Inc 2020)	Roluperidone 32mg, 64mg	Placebo	SCZ	Clinically stable	Double blinded RCT, phase III	PANSS negative symptom factor	514	12 weeks, followed by 40-week open-label extension	12-week study complete but unpublished. 40-week open label not yet complete.	USA, Eastern Europe	2017-2020	↔	No significant benefit of either dose at 12 weeks. See text for secondary outcomes (change in negative symptom factor -4.3 for roluperidone versus -3.5 for placebo)
EudraCT-2014-004878-42 MIN-101C03	(Davidson, Saoud et al. 2017)	Roluperidone 32mg, 64mg	Placebo	SCZ	Clinically stable with negative symptoms	Double blinded RCT, phase II	PANSS negative symptom factor	244	12 weeks	Complete	Eastern Europe	2014-2016	↔	Roluperidone 64mg vs placebo: mean difference -1.97, p ≤0.01 Roluperidone 32mg vs placebo: mean difference -1.54, p ≤0.05
NCT00861796 CYR-101C01	Unpublished, but reported in review by (Ebdrup, Bjørn H., Rasmussen et al. 2011).	Roluperidone 64mg	Placebo	SCZ or SZA	Total PANSS>60;CGI>4.	Double blinded RCT, phase II	Total PANSS	100	Primary end-point 4 weeks but treatment continued for 12 weeks	Complete	France	2008-2010	↔	Roluperidone vs placebo: mean difference -3, p=0.06

**Key to abbreviations and symbols:** SCZ: Schizophrenia SZA: Schizoaffective disorder RCT: Randomised controlled trial PANSS: Positive and negative symptom scale CGI: Clinical Global Impression ↑ : Better outcome in roluperidone group relative to comparator group (statistically significant) ↓ : Poorer outcome in roluperidone group relative to comparator group (statistically significant) ↔ : No statistically significant difference between roluperidone and comparator groups

**Table 10:** Clinical efficacy trials of ulotaront (SEP-363856) in schizophrenia

Identifier	Reference	Ulotaront dose(s)	Comparator group(s)	Indication	Patient group	Type of study, phase	Primary outcome measure	Number of patients enrolled	Length of treatment	Status	Location	Year	Results	
													Change in ulotaront group relative to comparator group	Effect size
NCT04109950		Ulotaront 25mg, 50mg, 75mg, 100mg	No comparator group	SCZ	Acute relapse,	Open-label extension study, phase III	Incidence of AEs and SAEs	555 planned	52 weeks	In progress	USA, Eastern Europe & Russia	2019-2022 (est.)	Due 2022	
NCT04115319		Ulotaront 50mg – 100mg	Quetiapine 400-800 mg	SCZ	Clinically stable	Double blinded flexible-dose RCT, phase III	Incidence of AEs and SAEs	300 planned	52 weeks	In progress	USA, Eastern Europe & Russia	2019-2022 (est.)	Due 2022	
NCT04092686		Ulotaront 75mg, 100mg	Placebo	SCZ	Acute relapse	Double blinded RCT, phase III	Change in PANSS total score	462 planned	6 weeks	In progress	USA, Eastern Europe & Russia	2019 – 2021 (est.)	Due 2021	
NCT04072354		Ulotaront 50mg, 75mg	Placebo	SCZ	Acute relapse	Double blinded RCT, phase III	Change in PANSS total score	525 planned	6 weeks	In progress	USA, Eastern Europe & Russia	2019-2021 (est.)	Due 2021	
NCT04038957		Adjunctive ulotaront 50-75mg + usual antipsychotic	No comparator group	SCZ	PANSS ≥ 70	Open label PET study phase I,	Change in dopamine synthesis capacity at week 2 using 18-F-DOPA	22 planned	2 weeks	In progress	UK	2019 – 2021 (est.)	Due 2021	
NCT04325737		Ulotaront (titrated from 50 to 100mg-cohort 1), ulotaront (titrated from 25 to 100mg-cohort 2)	Placebo	SCZ	Clinically stable	Phase I	Frequency of SEs and SAEs	32 planned	Cohort 1-14 days Cohort 2-17 days	In progress	Japan	2020-2021 (est)	Due May 2021	
NCT04369391		Ulotaront 150mg	Placebo, Moxifloxacin 400mg	SCZ	Clinically stable	Randomised, 3-period crossover study, phase I	Change from baseline QTc interval	72 planned	7 weeks	In progress	USA	June – November 2020 (est.)	Due November 2020	
NCT02970929	(Koblan, Kent et al. 2020)	Ulotaront 25mg, 50mg or 75mg	No comparator group	SCZ	Acute relapse	Open labelled extension study, phase II	Primary outcome: incidence of AEs and SAEs Secondary outcome: Change from extension-study baseline in PANSS total score	156	26 weeks	Completed	USA, Eastern Europe & Russia	2016-2018	N/A	Among patients who had initially been assigned to receive ulotaront and then continued treatment, the mean change from extension study baseline was -17.1. Among patients who had initially been assigned to receive placebo and then switched to open-label ulotaront in the extension study, the mean change from the extension study baseline was -27.9.
KiNCT02969382	(Koblan, Kent et al. 2020)	Ulotaront 50 -75mg	Placebo	SCZ	Acute relapse	Double blinded RCT, phase II	Change in PANSS total score	245	4 weeks	Completed	USA, Eastern Europe & Russia	2016-2018	↑	Ulotaront vs placebo: mean difference -7.5, p<0.01

**Key to abbreviations and symbols:** SCZ: Schizophrenia RCT: Randomised controlled trial AEs: Adverse events SAEs: Serious adverse events PANSS: Positive and negative symptom scale ↑ : Better outcome in ulotaront group relative to comparator group (statistically significant) ↓ : Poorer outcome in ulotaront group relative to comparator group (statistically significant) ↔ : No statistically significant difference between ulotaront and comparator groups

**Table 11:** Clinical efficacy trials of xanomeline in schizophrenia

Identifier	Reference	Xanomeline dose(s)	Comparator group (s)	Indication	Patient group	Type of study, phase	Primary outcome measure	Number of patients enrolled	Length of treatment	Status	Location	Year	Results	
													Change in xanomeline group relative to comparator group	Effect size
	(Shekhar, Potter et al. 2008)	Xanomeline 25mg – 75mg TDS	Placebo TDS	SCZ or SZA	Inpatients	Pilot study, double-blind, placebo-controlled RCT	PANSS, BPRS, CGI	20	4 weeks	Completed	USA	2008	↑	PANSS: mean difference -24.0, p<0.05 BPRS: mean difference -6.45, p<0.05 CGI: mean difference +1.1, p=0.94 List learning test: mean difference in score change +2.4, p<0.05 Story recall test: mean difference +1.4, p<0.05 Delayed memory test: mean difference + 22.6, p<0.05 No statistically significant difference between xanomeline and placebo groups for trail making test, continuous performance test, digit span, or domains of brief visuospatial memory test other than delayed memory. All mean differences relative to placebo. No multiplicity correction applied to cognition outcomes.
NCT03697252	(Brannan, Sawchak et al. 2021)	Xanomeline 50 – 125mg BD + trospium 20-30mg BD	Placebo BD	SCZ	Acute relapse requiring hospitalisation	Double blinded RCT, phase II	PANSS total	182	5 weeks	Completed	USA	2018 - 2019	↑	PANSS: LSMD -11.6, p<0.001 PANSS positive subscale: LSMD -3.2 (p<0.001) PANSS negative subscale: LSMD -2.3, p<0.001 All mean differences relative to placebo.

**Key to abbreviations and symbols:** SCZ: Schizophrenia SZA: Schizoaffective disorder PANSS: Positive and negative symptom scale BPRS: Brief psychiatric rating scale CGI: Clinical Global Impression RCT: Randomised controlled trial LSMD: Least squares mean difference BD: bis die: twice daily TDS: ter die sumendus: three times a day ↑ : Better outcome in xanomeline group relative to comparator group (statistically significant) ↓ : Poorer outcome in xanomeline group relative to comparator group (statistically significant) ↔ : No statistically significant difference between xanomeline and comparator groups

**Table 12:** Clinical efficacy trials of BI 409306 in schizophrenia

Identifier	Reference	BI 409306 dose(s)	Comparator group(s)	Indication	Patient group	Type of study, phase	Primary outcome measure	Number of patients enrolled	Length of treatment	Status	Location	Year	Results	
													Change in BI 409306 group relative to comparator group	Effect size
NCT03351244		BI 409306 ‘high dose’	BI 409306 ‘low dose’	Placebo	SCZ	Clinically stable	Double blinded RCT, phase II	Time to first relapse	264 planned	28 weeks	Terminated (disruption due to Covid-19)	North America, Europe, Asia	2017-2021	Study terminated	
NCT03230097		BI 409306	Placebo	APS	Age 16-30	Double blinded RCT, phase II	Time to remission from APS	50 planned	52 weeks	Terminated (disruption due to Covid-19)	North America, Europe, Asia	2017-2021	Study terminated	
NCT02281773	(Brown, Nakagome et al. 2019)	Adjunctive BI 409306 10mg, 25mg, 50mg, 100mg	Adjunctive placebo	SCZ	Clinically stable	Double blinded RCT, phase II	Change in MCCB score	518	12 weeks	Completed	North America, Europe, Asia	2014 - 2017	↔	BI 409306 10mg vs placebo: mean difference -1.2, p=0.126 BI 409306 25mg vs placebo: mean difference 0.3, p=0.733 BI 409306 50mg vs placebo: mean difference 0.3, p=0.699 BI 409306 100mg vs placebo: mean difference -0.6, p=0.42

**Key to abbreviations and symbols:** SCZ: Schizophrenia APS: Attenuated psychosis syndrome RCT: Randomised controlled trial MCCB: Matrics Consensus Cognitive Battery MATRICS: Measurement and Treatment Research to Improve Cognition in Schizophrenia PANSS: Positive and Negative syndrome scale ↑ : Better outcome in BI 409306 group relative to comparator group (statistically significant) ↓ : Poorer outcome in BI 409306 relative to comparator group (statistically significant) ↔ : No statistically significant difference between BI 409306 and comparator groups

**Table 13:** Clinical efficacy trials of BI 425809 in schizophrenia

Identifier	Reference	BI 425809 dose(s)	Comparator group(s)	Indication	Patient group	Type of study, phase	Primary outcome measure	Number of patients enrolled	Length of treatment	Status	Location	Year	Results	
													Change in BI 425809group relative to comparator group	Effect size
NCT04846868(CONNEX-1)		BI 425809 (dose not stated)	Placebo	SCZ	Clinically stable	Double blinded RCT, phase III, parallel group trial	Change in overall composite T-score of MATRICS CCB	586 planned	26 weeks	Recruiting	North America, South America, Asia & Europe	2021 – 2024 (est.)	Results due 2024	
NCT04846881 (CONNEX-2)		BI 425809 (dose not stated)	Placebo	SCZ	Clinically stable	Double blinded RCT, phase III, parallel group trial	Change in overall composite T-score of MATRICS CCB	586 planned	26 weeks	Recruiting	North America, South America, Asia & Europe (different sites to CONNEX-1)	2021 – 2024 (est.)	Results due 2024	
NCT04860830 (CONNEX-3)		BI 425809 (dose not stated)	Placebo	SCZ	Clinically stable	Double blinded RCT, phase III, parallel group trial	Change in overall composite T-score of MATRICS CCB	586 planned	26 weeks	Recruiting	North America, Asia & Europe	2021 – 2024 (est.)	Results due 2024	
NCT03859973		BI 425809 (dose not stated) + adjunctive computerised cognitive training	Placebo + adjunctive computerised cognitive training	SCZ	Clinically stable	Double blinded RCT, phase II	Change in overall composite T-score of MATRICS CCB	200 planned	12 weeks	Recruiting	North America, Europe & Australasia	2019-2022	Results due 2022	
NCT02832037	(Fleischhacker, Podhorna et al. 2021)	Adjunctive BI 425809 2mg, 5mg, 10mg, 25mg	Adjunctive placebo	SCZ	Clinically stable	Double blinded RCT, phase II	Change in MCCB score	509	12 weeks	Completed	North America, Europe & Asia	2016-2021	↑	BI 425809 10mg vs placebo: mean improvement 1.98, p<0.05 BI 425809 25mg vs placebo: mean improvement 1.73, p<0.05

**Key to abbreviations and symbols:** SCZ: Schizophrenia RCT: Randomised controlled trial AE: Adverse events MCCB: Measurement and Treatment Research to Improve Cognition Consensus Cognitive Battery ↑ : Better outcome in BI 425809 group relative to comparator group (statistically significant) ↓ : Poorer outcome in BI 425809 group relative to comparator group (statistically significant) ↔ : No statistically significant difference between BI 425809 and comparator groups

**Table 14:** Clinical efficacy trials of MK8189 in schizophrenia

Identifier	Reference	MK8189 dose(s)	Comparator group(s)	Indication	Patient group	Type of study, phase	Primary outcome measure	Number of patients enrolled	Length of treatment	Status	Location	Year	Results	
													Change in MK8189 group relative to comparator group	Effect size
NCT04624243		MK-8189 16mg, 24mg	Placebo	SCZ	Acute relapse	Double blinded RCT, phase II	Change in PANSS score	576	12 weeks	In progress	North America, Europe, Asia,	2020-2022 (est.)	Results due 2022	
NCT03055338		MK-8189 12mg	Risperidone 6mg, placebo	SCZ	Acute relapse	Double blinded randomised controlled parallel group trial, phase II	Change in PANSS score	224	4 weeks	Completed	USA	2017-2018	↔	MK8189 vs placebo: Mean difference -4.7, p=0.074 Risperidone vs placebo: mean difference -7.3, p=0.033

**Key to abbreviations and symbols:** SCZ: Schizophrenia RCT: Randomised controlled trial PANSS: Positive and Negative Syndrome Scale AE: Adverse events ↑ : Better outcome in MK-8189 group relative to comparator group (statistically significant) ↓ : Poorer outcome in MK-8189 group relative to comparator group (statistically significant) ↔ : No statistically significant difference between MK-8189 and comparator groups

**Table 15:** Summary table

Drug	Pharmacology	Type of evidence	Efficacy findings	Side-effect profile	Evaluation of evidence according to GRADE framework (Siemieniuk, Guyatt 2019)	Overall quality of evidence
**Cariprazine**	Partial dopamine D2/3 receptor agonist with very high D3 affinity	5 Short-term RCTs 1 Meta-analysis 2 Maintenance studies	Meta-analyses estimate mean difference in PANSS is between -6.23 and -9.71 over 6 weeks, similar to existing antipsychotics Potential benefit for negative symptoms in patients with persistent negative symptoms	Generally favourable side-effect profile with low risk of metabolic side effects 10% incidence of EPSEs	Inconsistency-NCT00404573 did not find significant difference between comparator groups	Moderate
**Brexpiprazole**	Partial dopamine agonist	5 Short-term RCTs 1 Maintenance study	Inconsistent results: 3 RCTs had positive findings, in 2 RCTs brexpiprazole failed to separate from placebo May improve social functioning	Lower incidence of akathisia than aripiprazole and cariprazine Minimal metabolic side effects	Inconsistency-3 RCTs had positive results at specific brexpiprazole doses	Moderate
**Brilaroxazine** **(RP5063)**	High-affinity D2, D3 and D4 receptor partial agonist	1 RCT	Brilaroxazine 15mg and 30mg groups had statistically significant reductions in PANSS compared to placebo in 1 RCT	EPSEs Akathisia Elevated liver enzymes No metabolic changes	Risk of bias- higher drop out rate in brilaroxazine 30mg group Imprecision-only 1 published RCT so far	Very low
**Lumateperone (ITI-007)**	High affinity 5HT2A and low-moderate D2 antagonist plus serotonin transporter inhibition	3 RCTs 1 Maintenance study	Inconsistent results: 2 RCTs had positive findings, 1 negative RCT Some results suggest improvement of social functioning and depressive symptoms	24% incidence of sedation 6.7% incidence of EPSEs No metabolic changes	Inconsistency- 2 of 3 RCTs had positive findings but only at specific lumateperone doses	Low
**F17464**	Very high-affinity D3 antagonist and 5-HT1A partial agonist	1 RCT	Efficacy of F17464 on overall and positive symptoms in 1 RCT, with some indication of benefit on cognitive symptoms	Insomnia (10.4%) Agitation (7.5%) Hyperlipidaemia (7.5%) Akathisia (4.5%)	Risk of bias- 19 subjects with protocol deviations Imprecision- only 1 published RCT so far	Very low
**Lu AF35700**	Dopamine D1, 5HT2A and 5-HT6 receptor antagonist	2 RCTs	No statistically significant difference between treatment and olanzapine/risperidone groups in 2 RCTs in patients with treatment resistant schizophrenia	Headache (8.2% in long term study) More data needed regarding cardiometabolic effects	Indirectness- no placebo-controlled studies to date, only tested in treatment resistance Imprecision- only 2 published RCTs so far	Low
**Pimavanserin (ACP-103)**	Inverse agonist on 5HT2A receptor, negligible action on D2	2 RCTs	No published data, a press release indicates improvement in negative symptom scores	No increased rates of EPSEs over placebo Potential to prolong QTc	Inconsistency- only 1 RCT with positive findings Imprecision- only 2 RCTs Risk of bias- results not formally published in peer published journal, only as press release on drug company website	Very low
**Roluperidone** **(MIN-101)**	5HT2A antagonist, no data indicating action on D2	3 RCTs	One RCT has shown a statistically significant improvement in negative symptoms, while two RCTs showed no significant difference in total symptoms between roluperidone and placebo groups	No increased rates of EPSEs over placebo	Inconsistency- only 1 RCT with positive findings	Low
**Ulotaront (SEP-363856)**	TAAR1 agonist with some affinity for 5HT1A receptors	1 RCT 1 Maintenance study	One RCT and one maintenance study so far have indicated its efficacy for total symptoms and positive and negative sub-scales	No increased rates of EPSEs No metabolic changes	Risk of bias- low placebo response in RCT Imprecision- only 1 published RCT so far	Very low
**Xanomeline** **(+ trospium)**	Muscarinic M1 and M4 agonist with no D2 affinity but functional dopamine antagonism	1 RCT 1 Pilot study	One phase 2 RCT indicates its efficacy on positive and negative symptoms Participants in the treatment arm of the pilot study showed improved positive, negative and cognitive symptoms	Gastrointestinal side effects No increased rates of EPSEs No metabolic effects	Risk of bias- cognitive outcomes not adjusted for multiplicity testing Imprecision- only 1 published RCT so far	Low
**BI 409306**	Phosphodiesterase 9A inhibitor	1 RCT	No statistically significant difference in cognition in treatment and placebo arms of 1 RCT	Visual symptoms (11.1%) Nasopharyngitis (3.2%) Nausea (2.6%) Dizziness (2.6%)	Imprecision- only 1 published RCT so far	Very low
**BI 425809**	Glycine transporter 1 inhibitor	1 RCT	Small statistically significant improvement in cognition in treatment arm of 1 RCT, though no improvement in functional outcomes	Headache (8-12%) Somnolence (2-6%) Gastrointestinal symptoms (2-11%) Anaemia (1-5%)	Imprecision- only 1 published RCT so far	Very low
**MK-8189**	Phosphodiesterase 10A inhibitor	1 RCT	No statistically significant difference between MK-8189 and placebo groups in 1 RCT	Tolerability results not yet published	Imprecision- only 1 published RCT so far Risk of bias- results not formally published in peer published journal, only on clinicaltrials.gov	Very low

## Highlights

- Several new agents reduce psychotic symptoms without direct action on D2 receptors
- Cariprazine, pimavanserin and roluperidone may treat negative symptoms
- Some data suggest roluperidone, xanomeline and BI425809 improve cognitive symptoms
- Partial D2 agonists and low D2 affinity drugs could benefit treatment resistance
- Novel agents have low rates of both extra-pyramidal and metabolic side effects

## Box 1 Pharmacology definitions

An **agonist** is a chemical that binds to and activates a receptor to result in a biological response. A **partial agonist** binds to and activates a receptor, but is not able to elicit the maximum possible response produced by a full agonist. In contrast, an **antagonist** blocks the action of the agonist, while an **inverse agonist** causes a pharmacological action opposite to that of the agonist. **Affinity** refers to the strength of the binding of the ligand to the receptor, or the likelihood that the ligand will bind to the receptor when it is in close proximity.

(Atack, Lavreysen 2010, Jackson 2010, Christopoulos, Stewart et al. 2010, Stewart, Sexton et al. 2010, Slifstein 2010).
